# Determinants of Polar versus Nematic Organization in Networks of Dynamic Microtubules and Mitotic Motors

**DOI:** 10.1016/j.cell.2018.09.029

**Published:** 2018-10-18

**Authors:** Johanna Roostalu, Jamie Rickman, Claire Thomas, François Nédélec, Thomas Surrey

**Affiliations:** 1 The Francis Crick Institute, 1 Midland Road, London NW1 1AT, UK; 2 European Molecular Biology Laboratory, Meyerhofstrasse 1, 69117 Heidelberg, Germany

**Keywords:** microtubule, cytoskeleton, kinesin, molecular motor, spindle assembly, self-organization, active network, *in vitro* reconsititution, computer simulation, Cytosim

## Abstract

During cell division, mitotic motors organize microtubules in the bipolar spindle into either polar arrays at the spindle poles or a “nematic” network of aligned microtubules at the spindle center. The reasons for the distinct self-organizing capacities of dynamic microtubules and different motors are not understood. Using *in vitro* reconstitution experiments and computer simulations, we show that the human mitotic motors kinesin-5 KIF11 and kinesin-14 HSET, despite opposite directionalities, can both organize dynamic microtubules into either polar or nematic networks. We show that in addition to the motor properties the natural asymmetry between microtubule plus- and minus-end growth critically contributes to the organizational potential of the motors. We identify two control parameters that capture system composition and kinetic properties and predict the outcome of microtubule network organization. These results elucidate a fundamental design principle of spindle bipolarity and establish general rules for active filament network organization.

## Highlights

• Kinesin-5 and kinesin-14 can produce nematic and polar microtubule networks
• Self-organizing network architecture depends on both motor and microtubule properties
• Two system-level control parameters determine which cytoskeletal network forms
• General rules explain the organizational capacities of mitotic motors in the spindle

## Graphical Abstract

**Figure:**
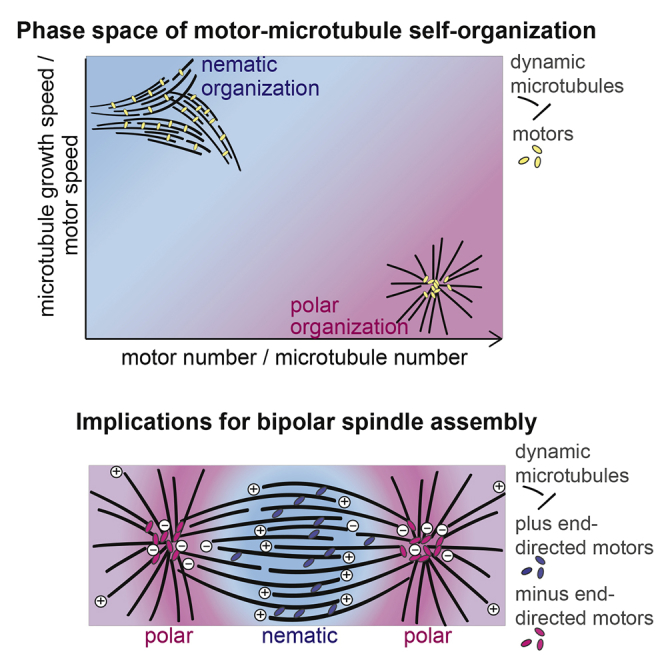

## Teaser

In vitro reconstitutions and computer simulations identify rules that govern microtubule network organization and reveal a design principle underlying bipolar spindle formation.

## Introduction

The internal organization of eukaryotic cells depends on cytoskeletal networks. Dynamic microtubules and actin filaments, motile crosslinkers, and other associated proteins drive active networks into a variety of organizational states required for distinct cell functions (Helmke et al., 2013, Sanchez and Feldman, 2017). Polarized microtubule networks serve as tracks for directional cargo transport during interphase (Kapitein and Hoogenraad, 2015, Keating and Borisy, 1999, Sanchez and Feldman, 2017). In contrast, in large cells of embryos and plants motors mediate the formation of arrays of aligned microtubules or actin filaments, causing global cytoplasmic flows to distribute nutrients and organelles (Ganguly et al., 2012, Goldstein et al., 2008, Monteith et al., 2016, Palacios and St Johnston, 2002). These networks consisting of aligned filaments of mixed-polarity are also called “nematic,” a term borrowed from liquid crystal terminology (Needleman and Dogic, 2017). How cells control the organization of active filament networks with different topologies is an open question.

During cell division, microtubule crosslinking motors organize microtubules into bipolar spindles, an architecture that is crucial for correct chromosome segregation. The role of motors is particularly evident in female meiosis, when the bipolar spindle self-organizes from randomly oriented microtubules nucleated locally in the vicinity of chromosomes (Heald et al., 1996). Minus-end-directed motors contribute to the formation of radial, polarized microtubule arrays with their minus ends focused at the spindle poles, and plus-end-directed motors are required to arrange nematic arrays of aligned microtubules with mixed-polarity in the spindle center (Brugués et al., 2012, Helmke et al., 2013, Kapoor, 2017). It is unclear why particular mitotic motors promote different organizational states. The critical determinants of filament self-organization are not known.

Biomimetic systems with limited sets of purified proteins have provided mechanistic insight that can be applied to intracellular networks. When microtubules were grown in the presence of artificial microtubule stabilizers, crosslinking motors produced locally contracting networks, leading to the formation of monopolar structures (asters) (Hentrich and Surrey, 2010, Nédélec et al., 1997, Surrey et al., 2001). Experimental and theoretical work suggested that such networks with polarity-sorted microtubules form when motors are sufficiently fast to reach microtubule ends and remain bound there, so that multiple microtubule ends can be brought together to form a stable radial array (Head et al., 2014, Nedelec and Surrey, 2001, Nédélec et al., 1997, Surrey et al., 2001, Torisawa et al., 2016). The minus-end-directed microtubule crosslinking motor kinesin-14 that contributes to spindle pole focusing in cells is one such motor that can form microtubule asters *in vitro* (Braun et al., 2017, Fink et al., 2009, Hentrich and Surrey, 2010, Kwon et al., 2008, Norris et al., 2018, Surrey et al., 2001).

Nematic networks of extensile bundles were observed *in vitro* when short, static microtubules were combined with purified artificial kinesin-1 clusters in the presence of crowding agents that promoted microtubule bundling (Henkin et al., 2014, Sanchez et al., 2012). Motors transported microtubules of mixed-polarity causing bundle extension and large-scale hydrodynamic flows. High microtubule concentrations and the crowding agent present in these experiments appeared to have changed the rules of self-organization. Whether motors can organize microtubules also into nematic networks under more physiological conditions is unknown. Kinesin-5 is the main plus-end-directed microtubule crosslinker that functions in the central region of the spindle and exerts outward forces in spindles (Blangy et al., 1995, Hagan and Yanagida, 1992, Hoyt et al., 1992, Kapitein et al., 2005, Miyamoto et al., 2004, Sawin et al., 1992, Sharp et al., 1999, Tanenbaum et al., 2008). This activity is consistent with its ability to slide individual pairs of anti-parallel microtubules apart *in vitro* (Hentrich and Surrey, 2010, Kapitein et al., 2005, Roostalu et al., 2011, van den Wildenberg et al., 2008). Yet, self-organization experiments with purified kinesin-5 and many microtubules have so far failed to reveal network organizations that correspond to this motor’s function in cells (Hentrich and Surrey, 2010, Torisawa et al., 2016).

Taken together, multiple parameters such as protein concentrations, kinetic motor properties, the degree of crowding, and potentially the dynamic properties of microtubules influence the organization of biological filament/motor systems. When trying to understand such a multi-dimensional organizational phase space, the question arises whether it is possible to identify a minimal set of critical determinants or control parameters that predict network organization. Identifying such control parameters can directly provide insight into complex system behavior.

To gain such a mechanistic understanding of microtubule/motor network organization, we explored the organizational capacity of human kinesin-5 and kinesin-14 in the presence of dynamic microtubules. In self-organization experiments with purified motors and microtubules with tunable plus- or minus-end dynamics, we show that both motors, despite opposite directionalities, can form either polar or nematic microtubule networks. We find that the normal asymmetry of microtubule growth plays an important role in determining a motor’s natural organizational capacity. Numerical computer simulations of active network organization identify two control parameters, each combining a motor and a microtubule property, that predict the outcome of network organization. Taken together, our results suggest a simple set of rules that explain bipolar spindle organization by mitotic motors and dynamic microtubules.

## Results

### KIF11 Organizes Dynamic Microtubules into Nematic Networks

**Figure 1:**
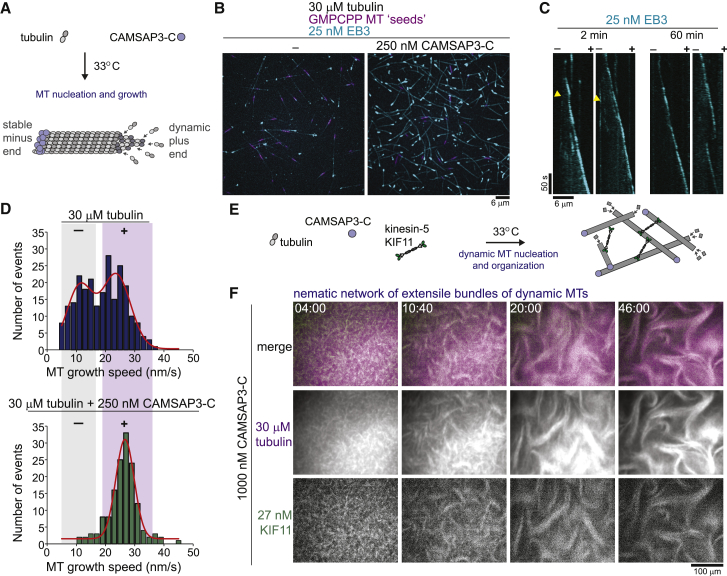
Self-Organization of Microtubules and Plus-End-Directed Motor KIF11 into Nematic Networks of Extensile Bundles (A) Scheme of CAMSAP3-C-mediated asymmetric microtubule growth. (B) Total internal reflection fluorescence (TIRF) microscopy images of 25 nM Alexa546-labeled SNAP-EB3 (Alexa546-EB3, cyan) tracking growing microtubule ends in the absence and presence of 250 nM mGFP-CAMSAP3-C at 30 μM tubulin. GMPCPP-stabilized microtubule “seeds” in magenta. Background subtracted maximum intensity projections of 25 frames imaged at 1/s 10 min after the start of microtubule nucleation are shown. (C) Kymographs showing microtubule plus-end growth using 25 nM Alexa546-EB3 in the presence of 250 nM mGFP-CAMSAP3-C starting 2 and 60 min after microtubule nucleation. Yellow arrowheads indicate non-growing minus-ends. (D) Microtubule growth speed distribution in the absence (top) and presence (bottom) of 250 nM mGFP-CAMSAP3-C at 30 μM tubulin. Number of growth episodes measured: without mGFP-CAMSAP3-C, 239; with mGFP-CAMSAP3-C, 148. Despite CAMSAP3-C not being restricted to microtubule minus-ends under these high CAMSAP3-C concentrations (Atherton et al., 2017), nucleated microtubules have asymmetric growth dynamics. (E) Scheme of motor/microtubule self-organization experiment. (F) Confocal fluorescence microscopy images showing time course of KIF11-mGFP-mediated (green) organization of a nematic network of extensile bundles of CF640R-labeled microtubules (magenta). Protein concentrations were: tubulin, 30 μM; mCherry-CAMSAP3-C, 1,000 nM; and KIF11-mGFP, 27 nM. Time in min:s. Temperature was 33°C. See also Figure S2 and Video S1.

**Figure S1:**
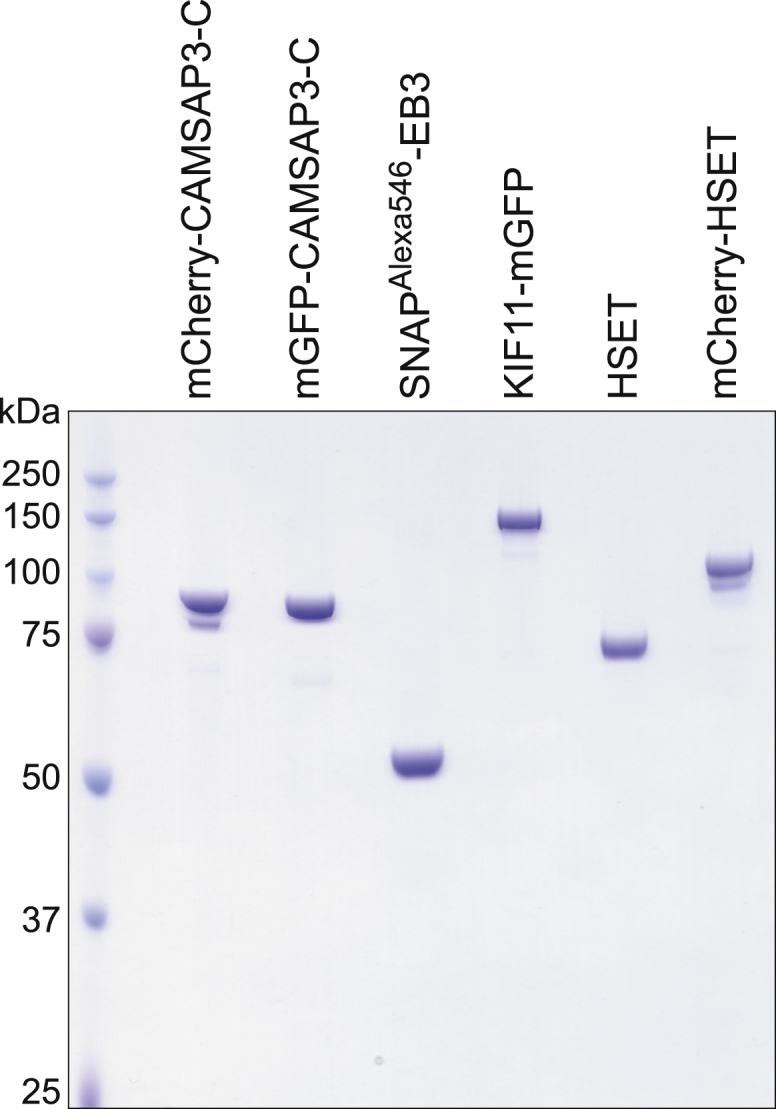
Coomassie-Stained SDS Gel with Purified Recombinant Proteins Used in This Study, Related to STAR Methods

To establish asymmetric microtubule growth dynamics *in vitro* and mimic the situation in the cell, we used a C-terminal fragment of the human microtubule minus-end stabilizer CAMSAP3 (CAMSAP3-C) (Figures 1A and S1) (Atherton et al., 2017, Hendershott and Vale, 2014, Jiang et al., 2014). Addition of CAMSAP3-C stimulated microtubule formation in pure tubulin solutions, as visualized by the end binding protein EB3 (Figures 1B and 1C) (Montenegro Gouveia et al., 2010). CAMSAP3-C suppressed minus-end dynamics and allowed the plus-ends of the nucleated microtubules to grow for at least an hour (Figures 1C and 1D).

**Figure S2:**
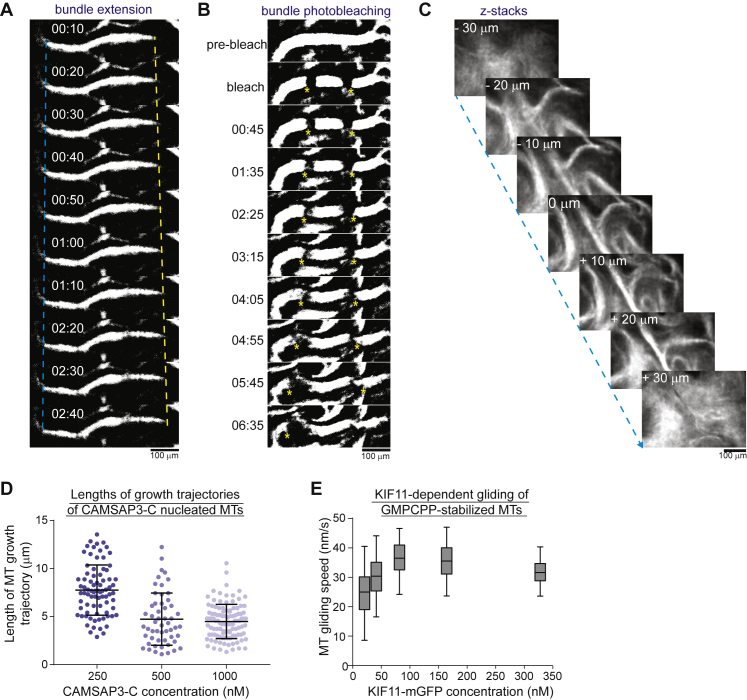
KIF11-Dependent Organization of Nematic Networks of Extensile Bundles, Related to Figure 1 (A) Binary confocal fluorescence microscopy images showing at high time-resolution the time course of dynamic fluorescent microtubule bundle extension within a nematic network organized by KIF11. (B) Binary confocal fluorescence microscopy images showing the separation of two photo-bleached marks (yellow asterisks) in an extensile bundle within a nematic network organized by KIF11. Binary images (after background subtraction and thresholding) of the microtubule channel are presented to enhance the visibility of distinct network parts. Time is in min:s. (C) Nematic networks organized by KIF11 are three-dimensional as revealed by confocal imaging of different focal planes in the flow chamber. Experiments presented in A - C were performed in the presence of 27 nM KIF11-mGFP, 30 μM tubulin, and 1000 nM mCherry-CAMSAP3-C. (D) Scatterplot depicting microtubule growth episode lengths at different CAMSAP3-C concentrations. Number of growth episodes measured at different mGFP-CAMSAP3-C concentrations: 250 nM – 80, 500 nM – 54, 1000 nM – 107. Horizontal lines indicate the mean and the standard deviation. (E) Box-and-whiskers plot depicting the dependence of KIF11-driven microtubule gliding speeds on the KIF11-mGFP concentration used to immobilise the motor on the glass surface for gliding assays with GMPCPP-stabilized microtubules. The measured speeds agree with previously reported speeds of metazoan kinesin-5 motors (Cole et al., 1994, Hentrich and Surrey, 2010, Kapitein et al., 2005, Krzysiak et al., 2006, Ma et al., 2011, Sawin et al., 1992, van den Wildenberg et al., 2008). The boxes extend from 25^th^ to 75^th^ percentiles, the whiskers extend from 5^th^ to 95^th^ percentiles, and the mean value is plotted as a line in the middle of the box. Number of gliding episodes measured at different KIF11-mGFP concentrations: 20.5 nM – 77, 41 nM – 87, 82 nM – 150, 164 nM – 128, 328 nM – 105. All experiments were carried out in self-organization buffer at 33°C.

We next investigated how purified human plus-end-directed microtubule crosslinking kinesin-5 KIF11 (Ferenz et al., 2010) organizes free microtubules with such asymmetric growth properties. We mixed fluorescently labeled tubulin and motors and initiated microtubule nucleation by a temperature shift (Figure 1E). Within minutes, KIF11 produced dynamic networks of aligned microtubules, generating persistent large-scale wave-like movements (Figure 1F; Video S1). This network differed from unstructured or contractile networks assembled by purified kinesin-5 in the presence of microtubule-stabilizing agents observed previously (Hentrich and Surrey, 2010, Torisawa et al., 2016). Instead, the aligned microtubule bundles appeared to be locally extensile as shown by increasing bundle lengths or increasing distances between photo-bleached bundle segments over time (Figures S2A and S2B). This suggests that KIF11-dependent anti-parallel sliding drives the organization and motion of these three-dimensional microtubule networks (Figure S2C). Together, these results demonstrate that nematic networks of extensile bundles can be generated by a natural mitotic kinesin and dynamic microtubules with a broad length distribution (Figure S2D). This formation of a nematic network by KIF11 reconstitutes its function in the central part of spindles.

Video S1. Plus-end-Directed Kinesin-5 KIF11 (Green) Organizes CAMSAP3-C Nucleated Dynamic Microtubules (Magenta) into Nematic Networks of Extensile Bundles, Related to Figure 1 Protein concentrations were: tubulin - 30 μM, mCherry-CAMSAP3-C - 1000 nM, and KIF11-mGFP – 27 nM. Time is in min:s. Imaging was carried out at 33°C.

**Figure 2:**
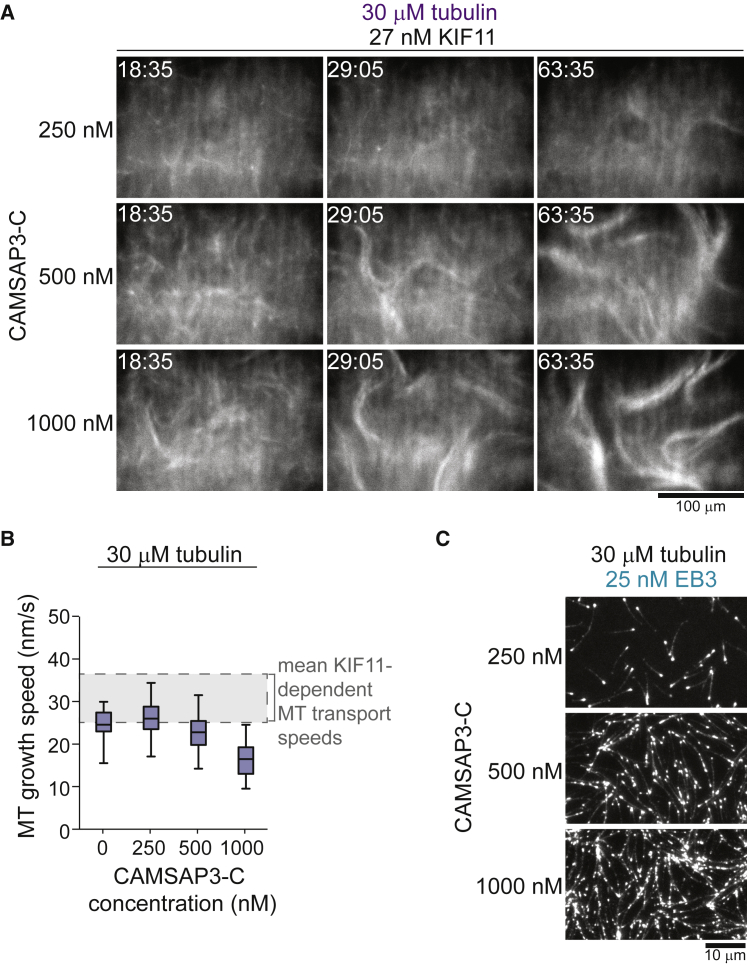
CAMSAP3-C Concentration Influences Microtubule Self-Organization by Affecting Microtubule Growth Speed and Density (A) Confocal fluorescence microscopy images showing a time course of KIF11-mGFP-dependent organization of CF640R-labeled microtubules at different mCherry-CAMSAP3-C concentrations. Tubulin and KIF11 are present at 30 μM and 27 nM, respectively. Time in min:s. (B) Box-and-whiskers plot depicting microtubule plus-end growth speeds at different CAMSAP3-C concentrations. The boxes extend from 25^th^ to 75^th^ percentiles, the whiskers extend from 5^th^ to 95^th^ percentiles, and the mean value is plotted as a line in the middle of the box. Number of plus-end growth episodes measured at different mGFP-CAMSAP3-C concentrations: 0 nM, 61; 250 nM, 148; 500 nM, 72; 1,000 nM, 186. The same source data has been used for the 0 nM and 250 nM condition as for Figure 1D. The shaded area indicates the typical range of KIF11-dependent microtubule transport speeds as estimated from microtubule gliding assays in the same buffer (Figure S2E). (C) TIRF microscopy images of 25 nM Alexa546-EB3 tracking growing microtubule ends showing enhanced microtubule formation at increasing mGFP-CAMSAP3-C concentrations at 30 μM tubulin (imaged at 2 min 20 s after initiating microtubule nucleation). Temperature was 33°C.

Reducing the CAMSAP3-C concentration lowered the efficiency of nematic network formation by dynamic microtubules and KIF11 (Figure 2A). To understand the reason behind this tendency, we measured the microtubule growth and KIF11 speeds under conditions similar to the self-organization experiments. Plus-end growth speeds decreased slightly with increasing CAMSAP3-C concentration (Figure 2B), probably as a consequence of soluble tubulin depletion at increased nucleation efficiencies resulting in more microtubule polymer (Figure 2C). The KIF11 speed was slightly higher than the microtubule growth speeds (Figures 2B, gray range, and S2E). Nevertheless, KIF11 did not form asters in self-organization experiments by coalescing microtubule plus-ends (Figure 2C) but distributed uniformly throughout the nematic network at the higher CAMSAP3-C concentrations (Figure 1F). These results indicate that increased microtubule densities present at higher CAMSAP3-C concentrations may hinder aster formation by promoting bundling of unsorted microtubules despite the motors moving slightly faster than microtubules grow.

### KIF11 Can Also Organize Dynamic Microtubules into Polar Networks

**Figure 3:**
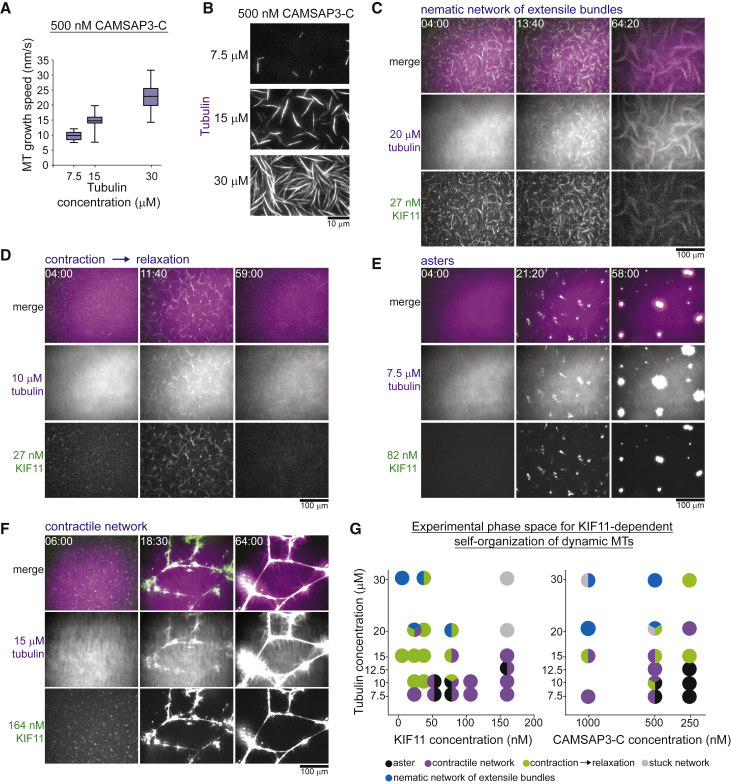
Tubulin and KIF11 Concentrations Influence Microtubule Network Organization (A) Box-and-whiskers plot depicting microtubule plus-end growth speeds at different CAMSAP3-C concentrations. Number of plus-end growth episodes measured at different tubulin concentrations: 7.5 μM, 31; 15 μM, 17; 30 μM, 72. The boxes extend from 25th to 75th percentiles, the whiskers extend from 5th to 95th percentiles, and the mean value is plotted as a line in the middle of the box. The same data is plotted for the 30 μM condition at 500 nM mGFP-CAMSAP3-C as for Figure 2A. (B) TIRF microscopy images of CF640R-labeled microtubules showing enhanced microtubule formation at increasing tubulin concentrations in the presence of 500 nM mGFP-CAMSAP3-C (imaged 2 min 20 s after initiating microtubule nucleation). (C–F) Confocal fluorescence microscopy images showing the time course of KIF11-mediated organization of different types of networks at the following respective concentrations for tubulin and KIF11: 20 μM and 27 nM (C), 10 μM and 27 nM (D), 7.5 μM and 82 nM (E), and 15 μM and 164 nM (F). The mCherry-CAMSAP3-C concentration was always 500 nM. Time in min:s. (G) Organizational phase spaces summarizing the different experimental outcomes of KIF11-mediated microtubule network organization as a function of KIF11 and tubulin concentrations (left) and as a function of CAMSAP3-C and tubulin concentrations (right). Both plots pool the outcomes of the same 60 self-organization reactions. Temperature was 33°C. See also Videos S2 and S3.

To understand KIF11-dependent network assembly, we explored the experimental phase space of microtubule organization. Keeping the CAMSAP3-C concentration fixed (500 nM), we lowered the tubulin concentration to reduce the microtubule plus-end growth speed (Figure 3A) and the amount of polymerized tubulin (Figure 3B). Networks of extensile bundles still formed, but more slowly (Figure 3C). At further reduced tubulin concentration, initially bundled networks appeared to form and began to polarity-sort and contract, as indicated by the local increase of the microtubule and KIF11 intensities (Figure 3D; Video S2). However, over time these foci dissolved accompanied by the relaxation and disengagement of the network as indicated by a rather diffuse distribution of microtubules and motors. Remarkably, when the tubulin concentration was further lowered and the KIF11 concentration increased, isolated asters formed. Strong motor accumulation in the aster center indicated that microtubules were polarity-sorted with their plus-ends focused inward (Figure 3E; Video S3). Increasing both the KIF11 and the tubulin concentration produced tense interconnected contractile networks (Figure 3F).

Video S2. Plus-end-Directed Kinesin-5 KIF11 (Green) Organizes CAMSAP3-C Nucleated Dynamic Microtubules (Magenta) into Initially Contracting Networks that Subsequently Undergo Relaxation, Related to Figure 3 Protein concentrations were: tubulin - 10 μM, mCherry-CAMSAP3-C - 500 nM, and KIF11-mGFP – 27 nM. Time is in min:s. Imaging was carried out at 33°C.

Video S3. Plus-end-Directed Kinesin-5 KIF11 (green) Organizes CAMSAP3-C Nucleated Dynamic Microtubules (Magenta) into Locally Contractile Polar Networks or Asters, Related to Figure 3 Protein concentrations were: tubulin – 7.5 μM, mCherry-CAMSAP3-C - 500 nM, and KIF11-mGFP – 82 nM. Time is in min:s. Imaging was carried out at 33°C.

These results demonstrate that the same motor can form either locally extensile nematic or locally contractile polar networks simply depending on the protein composition of the system. High tubulin and low KIF11 concentrations promote nematic network organization (Figure 3G, left, blue), in agreement with high concentrations of static microtubules being a requirement for nematic network formation in the presence of crowding agents (Henkin et al., 2014, Sanchez et al., 2012). In contrast, increased KIF11 concentrations at low tubulin concentrations produced isolated asters (Figure 3G, left, black) or globally contracting networks (Figure 3G, left, purple). The specific outcome of self-organization also depends critically on the CAMSAP3-C concentration (Figure 3G,right), which affects both the microtubule density and indirectly their growth speed (Figures 2B and 2C). Taken together, higher microtubule concentrations and higher growth speeds promote nematic network formation (Figure 3G, right, blue), whereas slow growth speeds and sparse microtubules promote polar network organization (Figure 3G, right, black).

### Competition between Motor Crosslinks at the Ends and Along the Sides of Microtubules Determines Network Organization

**Figure S3:**
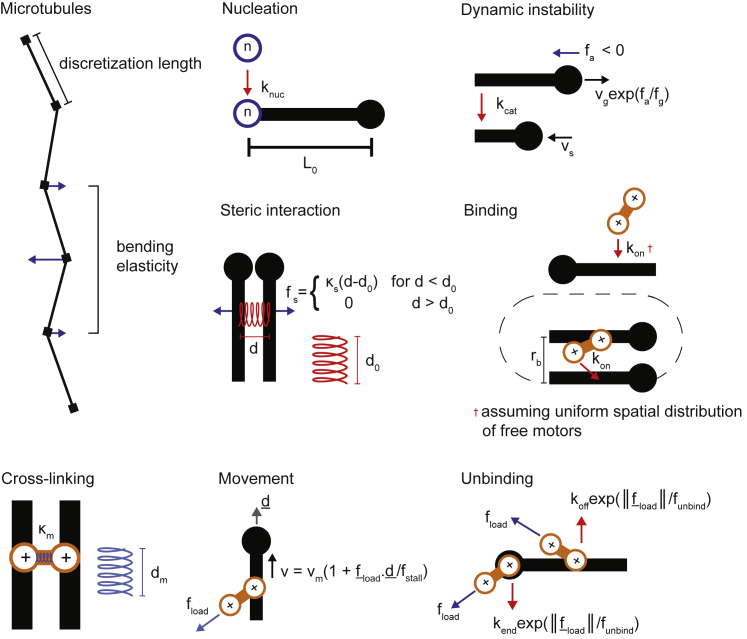
Schematic Showing the Elements of Cytosim, Related to STAR Methods

To understand mechanistically how dynamic microtubules and crosslinking motors drive network self-organization, we investigated the organizational phase space with three-dimensional numerical simulations using Cytosim (Nedelec and Foethke, 2007) (Figure S3). Global network contraction has been studied extensively (Alvarado et al., 2017, Belmonte et al., 2017, Foster et al., 2015, Letort et al., 2015, Stam et al., 2017, Torisawa et al., 2016). We therefore focused on the conditions that lead to nematic networks or asters, which show opposite characteristics regarding local extension versus contractility, and the degree of microtubule polarity-sorting. The simulation space was a thin three-dimensional box (40 × 40 × 0.4 μm). Microtubules were modeled as elastic rods with a static minus- and dynamic plus-end. They nucleated stochastically and grew to an average length of 2.5 μm before undergoing catastrophe after which they shrunk and vanished. New microtubules were nucleated by a fixed amount of nucleators, maintaining their number at steady state. Soft-core steric interactions were implemented between microtubules. Kinesin-5-like crosslinking motors bound stochastically up to two microtubules simultaneously and walked processively toward microtubule plus-ends, unbinding stochastically. Motors that reached the ends did not unbind instantaneously but dwelled there for a finite time. Microtubule growth and motor speed, key parameters of the model, were based on our experimental measurements, other parameters were based on previously measured values (Table S1).

**Figure 4:**
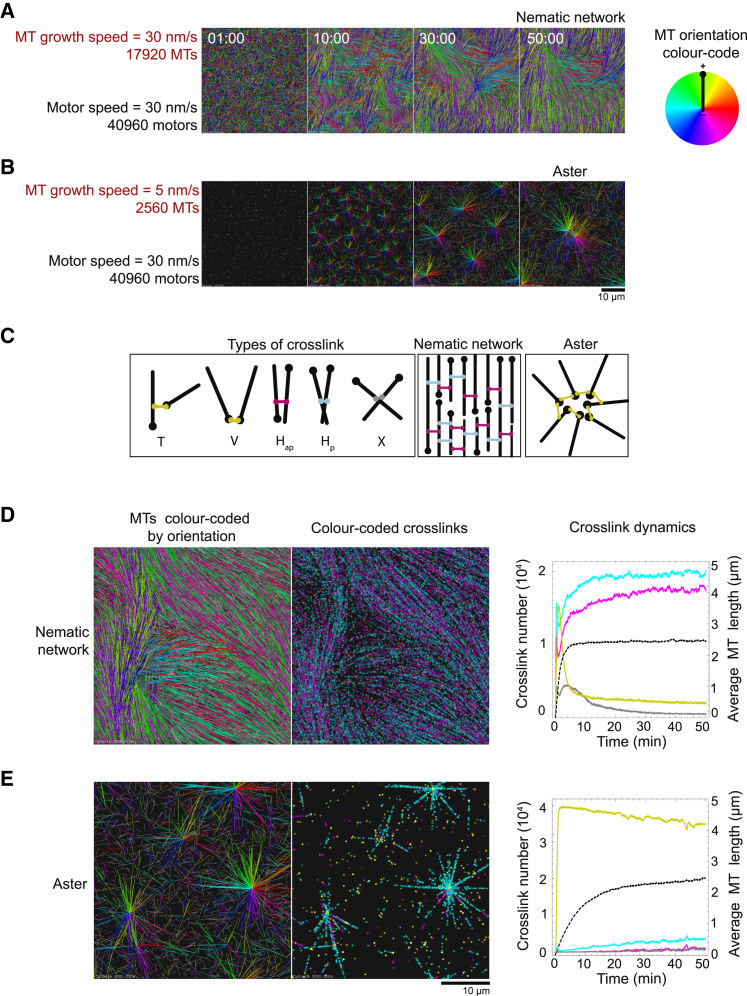
Computer Simulations Reveal Two Distinct Microtubule/Motor Organizational States (A) Snapshots showing the evolution of a nematic microtubule network. Simulated time in min:s. All simulation images are 3D projections of a snapshot onto the x-y plane. Colors indicate microtubule orientation (code: right). For visual clarity unconnected microtubules bearing no crosslinking motors are displayed in gray. (B) Snapshots showing the evolution of asters. Simulated time in min:s. (C) Schematic defining 5 different ways in which a motor can crosslink two microtubules (left). Schematic representations of the organization of microtubules and the composition of crosslinks in the nematic network state and the aster state (middle and right). (D) Final snapshot of a nematic network showing only microtubules (left) and only motor crosslinks color-coded according to their type as in (C) (middle). Plot showing the time courses of different populations of motor crosslinks (colored lines, color-coded as in C) and the average microtubule length (black dashed line) for the nematic network (right). (E) Final snapshot of an aster state showing only microtubules (left) and only motor crosslinks color-coded according to their type as in (C) (middle). Plot showing the time courses of different populations of motor crosslinks (colored lines, color-coded as in C) and the average microtubule length (black dashed line) for the aster state (right). Simulation parameters are the same as in (A) and (B) for the nematic network and the asters, respectively. See also Figure S4 and Videos S4 and S5.

Simulations allowed us to separate the effects of microtubule growth speed and microtubule number that vary concurrently in experiments when the tubulin or CAMSAP3-C concentration is changed. Systematically varying key parameters revealed a rich phase space with two distinct stable network organizations; a nematic network of aligned, mixed-polarity microtubule domains (Figure 4A) and a polar state of isolated asters with interconnected microtubule plus-ends (Figure 4B). The mechanistic principles behind these transitions can be understood from the different types of motor crosslinks characterizing each state (Figure 4C).

**Figure S4:**
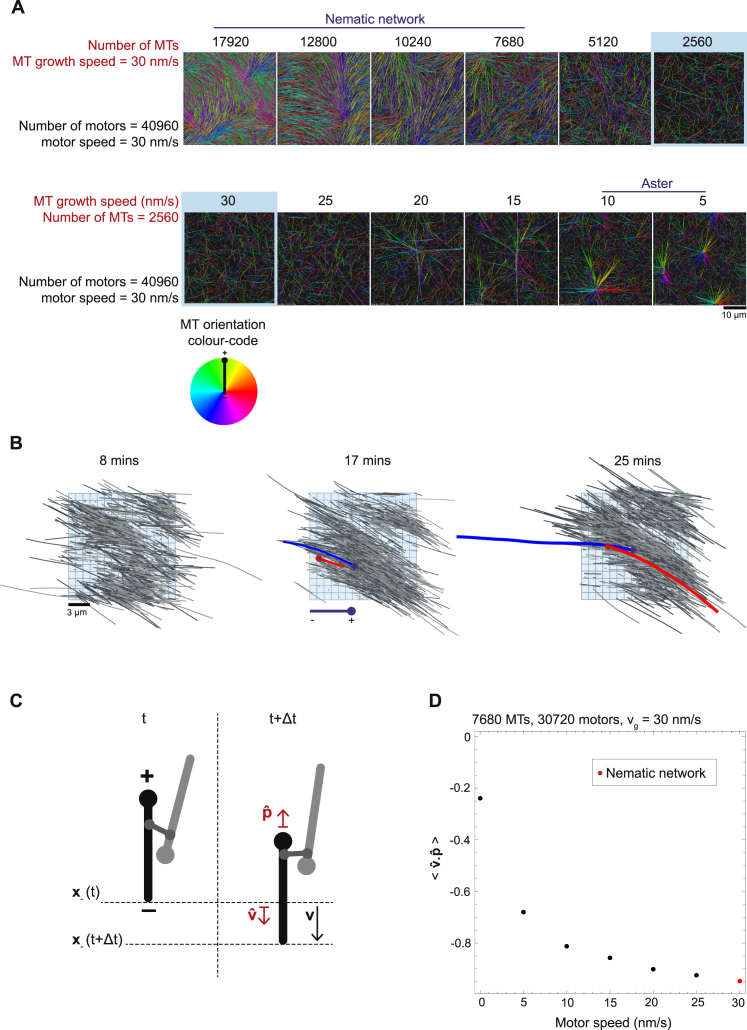
The Nematic Network State Exhibits Extensile Behavior, Related to Figure 4 There is a gradual transition between the nematic and aster state upon decreasing microtubule number and microtubule growth speed. (A) Snapshots of final simulation outcomes as parameters are systematically varied. The number of microtubules (top) and the microtubule growth speed (bottom) are varied while holding all other parameters constant. The colored blue border indicates simulations with the same parameter values. The type of organizational state is labeled above the simulation snapshot. All simulation images are three-dimensional projections of a snapshot onto the x-y plane. Colors indicate microtubule orientation. Color code: below, left. For visual clarity unconnected microtubules bearing no crosslinking motors are displayed in gray. See Table S1 for simulation parameters if not shown. (B) Time-course showing simulation snapshots of a nematic network (taken from Video S4). An aligned domain of microtubules is isolated from the network and shown alone so that the extension of the domain can be clearly seen. Microtubules within this domain are selected on the basis that any point along their length falls within the volume described by (−5 < x < 10 μm, −10 < y < 5 μm, −0.2 < z < 0.2 μm) (shown by a colored blue box, the origin is located at the center of the simulation space) and their long axis is oriented at an angle of −93° < θ < −53° with respect to the vertical. Microtubules colored in light and dark gray point in opposite directions. The trajectories of two oppositely oriented microtubules are highlighted (blue and red). The distance between their static minus ends increases due to anti-parallel sliding by motors while their plus-ends grow. Overall anti-parallel sliding results in the narrowing and lengthening of the entire domain along its long axis over time. (C) Schematic illustration of the calculation of the parameter $\hat{v}\cdot \hat{p}$ (STAR Methods) for a single microtubule (black) driven backward via a crosslinking motor connecting it to an anti-parallel microtubule (gray). (D) A plot showing the average value $\hat{v}\cdot \hat{p}$ (STAR Methods) for a range of different motor speeds. Each point represents one simulation and the final point (red) represents the nematic network state. The increasing negative value of $\hat{v}\cdot \hat{p}$ with motor speed demonstrates that microtubules are being continuously transported backward by motors, which drives the extension of the aligned microtubule domains.

The nematic network state occupied a parameter regime corresponding experimentally to a high tubulin concentration. High microtubule numbers promoted steric interactions leading to microtubule alignment (Figures 4D, left, and S4A, top) and the formation of side-side motor crosslinks connecting parallel (H_p_ links) and anti-parallel microtubules (H_ap_ links). In this regime, microtubule growth speed was comparable to the motor speed; motors did not efficiently reach microtubule plus-ends but dwelled on the microtubules’ sides. Equal numbers of H_p_ and H_ap_ crosslinks therefore dominate in the nematic network state (Figure 4D, middle, right). Both H_p_ and H_ap_ crosslinks are motile, but only H_ap_ links contribute to relative microtubule sliding and the extension of aligned microtubule domains (Figures S4B–S4D; Video S4) similar to the nematic networks of extensile bundles observed experimentally (Figures 1F and 2C; Video S1).

Video S4. Simulation of a Nematic Network, Related to Figure 4 Three-dimensional projections onto the x-y plane are shown. (Left) Microtubules only are displayed, color-coded according to their orientation (see Figure 4A). (Right) Only motor crosslinks are shown color-coded according to their type (see Figure 4C). Parameter values, as in Figure 4A: 17920 microtubules, 40960 motors, microtubule growth speed = 30 nm/s, motor speed = 30 nm/s. For all other parameter values see Table S1. Simulated time is in min:s.

The aster state occupied a parameter regime corresponding experimentally to a low tubulin concentration. When the number of microtubules was lowered and the microtubule growth speed was decreased to 3–6 times below the motor speed, isolated asters formed with motors accumulating at their centers (Figure 4E, left, and S4A, bottom; Video S5) like in the experiments (Figure 3E; Video S3). At slow microtubule growth speeds, motors efficiently reached microtubule plus-ends. End-side crosslinks (T links) that transform into end-end crosslinks (V links) dominate this regime (Figure 4E, middle, right). At low microtubule numbers and with enough motors, microtubule plus-ends are connected via V links into isolated asters (Figure 4E, left, middle) (Head et al., 2014, Nedelec and Surrey, 2001, Nédélec et al., 1997). V links are static and accumulate at the center of the asters (Figure 4E, middle) and H_p_ links move inward on the asters’ spokes (Video S5). The gradual transition between the nematic and polar states (Figure S4A) and their respective parameter regimes are in good agreement with the experimental phase space (Figure 3G, left). The crosslink dynamics also demonstrate that a stable network topology is established within the simulated time (Figures 4D and 4E, right).

Video S5. Simulation of Asters, Related to Figure 4 Three-dimensional projections onto the x-y plane are shown. (Left) Microtubules only are displayed, color-coded according to their orientation (see Figure 4A). (Right) Only motor crosslinks are shown color-coded according to their type (see Figure 4C). Parameter values, as in Figure 4B: 2560 microtubules, 40960 motors, microtubule growth speed = 5 nm/s, motor speed = 30 nm/s. For all other parameter values see Table S1. Simulated time is in min:s.

### Dimensionality Reduction of Parameter Space Reveals Two Control Parameters

**Figure S5:**
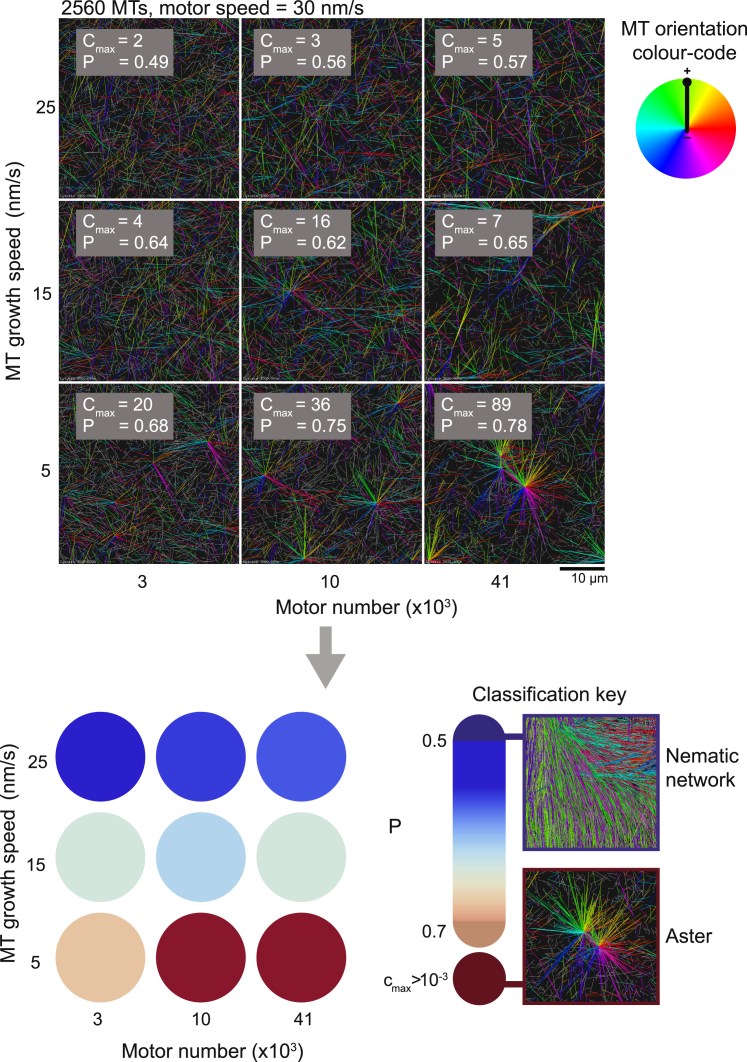
Visualization of the Phase Space of Simulated Microtubule-Motor Networks by Classifying and Color-Coding the Network Types, Related to Figure 5 Bespoke order parameters reveal a gradual transition between nematic and polar states. Final snapshots from 9 of the simulations from the phase space in Figure 5A, left, are shown. All simulation images are three-dimensional projections of a snapshot onto the x-y plane. Colors indicate microtubule orientation. Color code: right. For visual clarity unconnected microtubules bearing no crosslinking motors are displayed in gray. Each simulation outcome is classified using two bespoke parameters c_max_ and P (STAR Methods). The values of C_max_ and P are shown overlaid on the snapshots, where C_max_ = c_max_ x microtubule number. The classified state is displayed in the phase space below as a colored circle according to the classification key (left of the phase space). Due to low microtubule numbers in this parameter scan the nematic states (blue circles) show less alignment than the nematic network of aligned microtubule domains described in Figures 4A and 4D. However, the degree of polarity-sorting, captured by P, is similar in both cases.

What are the control parameters that govern the composition of different crosslinks in the network and consequently its topology? We screened the parameter space and classified the outcome of self-organization according to two bespoke criteria; an aster strength parameter, c_max_, and a polarity-sorting parameter P, based on the composition of motor crosslinks in the network and its connectivity. Color-coding the network types allowed us to visualize the different organizational states in the phase space (Figure S5).

**Figure 5:**
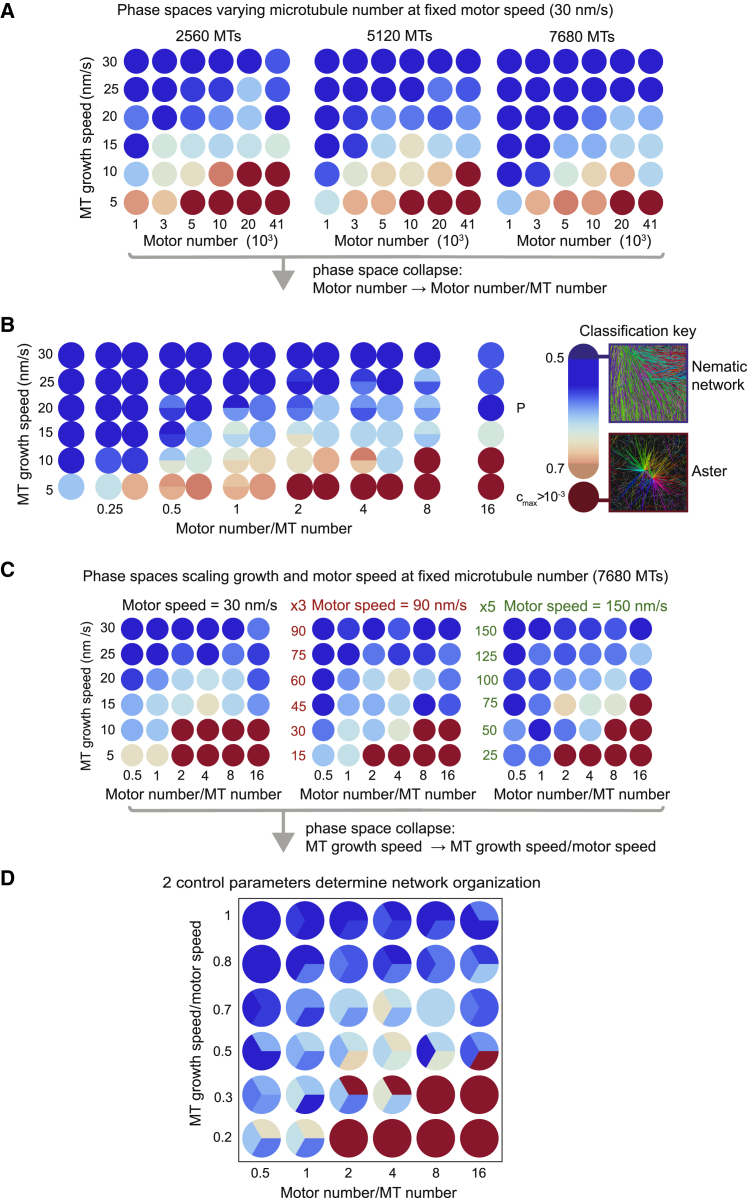
Computational Exploration of the Multi-Dimensional Parameter Space of Microtubule/Motor Networks Reveals Critical Parameters Driving Active Network Organization (A) Three phase spaces showing the organizational state of the network as a function of microtubule growth speed and motor number at three different numbers of microtubules. Simulation outcomes are classified (Figure S5; STAR Methods) and color-coded (see “Classification key”). Each circle represents one simulation. (B) Phase spaces in (A) can be collapsed onto a single space by plotting the classified states as a function of growth speed and the number of motors per microtubule. Where simulations are coincident in the collapsed phase space the circle is divided between them. (C) Three collapsed phase spaces for three different motor and microtubule speed scalings. Speeds are increased by a factor of 3 (middle) and 5 (right). (D) Phase spaces in (C) can be collapsed onto a single space by plotting the classified states as a function of the ratio of growth speed to motor speed and the number of motors per microtubule. For all simulations see Table S1 for parameter values if not shown. See also Figure S6.

First, we varied the microtubule growth speed and the number of motors at three different numbers of microtubules (keeping motor speed constant), producing three planar sections through the multi-dimensional parameter space (Figure 5A). Nematic networks (blue) formed at high microtubule growth speeds and asters (red) formed at low microtubule growth speeds and high motor numbers. A gradual transition zone (blue to orange) separated the nematic and polar states (Figures 5A and S5).

The boundary between the different states in each phase space shifted systematically with increasing microtubule number, favoring the nematic states at the expense of the polarity-sorted asters (Figure 5A). The three phase spaces could be collapsed on top of each other by plotting the same data as a function of microtubule growth speed and a combined parameter, i.e., the ratio of the motor number per microtubule number (N_Mot_/N_MT_) (Figure 5B). This suggests that the number of motors per microtubule is a control parameter for network formation.

**Figure S6:**
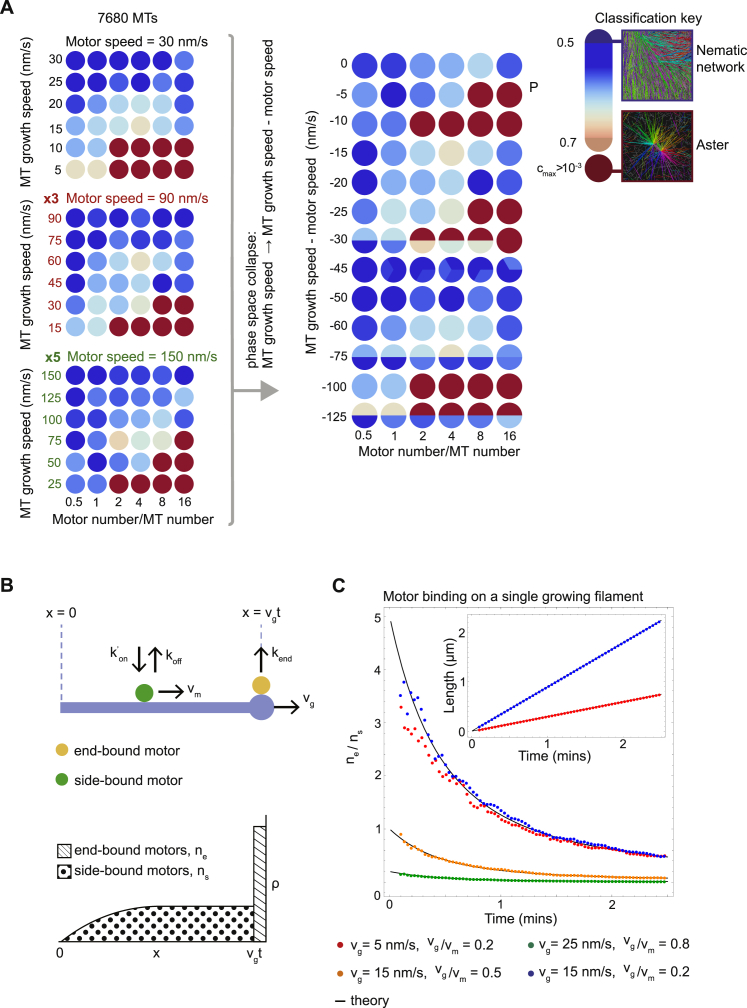
The Ratio of Motor Speed v_m_ to Microtubule Growth Speed v_g_ Determines the Ratio of End-Bound Motors to Side-Bound Motors on a Single Microtubule, Related to Figure 5 (A) Failed attempt to collapse the three phase spaces in Figure 5C plotting here the *difference* in motor speed and growth speed against motor number per microtubule. Different colors indicate different types of network according to the classification key shown on the right (STAR Methods). Where simulations are coincident in the collapsed phase space the circle is divided between them. y axis is not shown to scale. Compare with the successful phase space collapse using the ratio of motor speed and growth speed in Figure 5D. (B) (Top) Schematic representation of the single filament model showing binding and unbinding kinetics of a motor on a microtubule. Binding and unbinding of motors from the side of the microtubule occurs at rates k^’^_on_ and k_off_ respectively. Motors move deterministically at speed v_m_ to the plus-end that is growing at speed v_g_. Motors unbind from the plus-end at rate k_end_. (Bottom) Example motor density profile. Dotted area represents the total number of motors on the side, n_s_, and lined area represents the total number of motors at the plus-end, n_e_. (C) Time evolution of the ratio of end-bound motors to side-bound motors on a single growing microtubule for different pairs of parameters v_m_ and v_g_. Colored points represent average results from 4 simulations (see Table S1 for parameter values) and black lines correspond to theory (Equation S5). Inset shows the average length of the microtubules over the same period for two parameter sets. For the same ratio but different magnitudes of v_m_ and v_g_ (red circles, blue crosses), the ratio of side-bound to end-bound motors will be the same at a given time point in the microtubule’s lifetime, although the lengths of the microtubules will differ at this time. This provides a mechanistic explanation as to why the ratio of motor speed to microtubule growth speed is a control parameter in our model; it captures the spatial distribution of motor crosslinks on microtubules.

To analyze how motor speed relative to microtubule growth speed determines network organization, we explored the structure of collapsed phase spaces for three motor speeds. Scaling the microtubule growth and motor speeds by a factor of 3 and 5 (Figure 5C; Table S1) resulted in similar phase spaces that could be collapsed together by using the ratio—but not the difference (Figure S6A)—of the microtubule growth speed per motor speed (v_g_/v_m_) as a second combined parameter (Figure 5D). One can show theoretically that the ratio of microtubule growth speed to motor speed determines the ratio of end-bound motors to side-bound motors on a single filament (Figures S6B and S6C). Extrapolating this to microtubule/motor networks suggests that the parameter v_g_/v_m_ controls the spatial distribution of different motor crosslinks. When v_g_/v_m_ is low, there are many end-end crosslinks relative to side-side crosslinks, favoring the formation of asters (Figures 4B, 4D, and 5D). The opposite is true when v_g_/v_m_ is high, which leads to nematic states that do not polarity-sort (Figures 4A, 4D, and 5D).

The single filament theory also shows that motor accumulation at plus-ends is strongest when microtubules are short (Figure S6C). This explains why the population of V links peaks at early times and is depleted in favor of H_p_ and H_ap_ links as the average microtubule length increases (Figures 4D and 4E, right).

The tilted boundaries between nematic and polarity-sorted states in the phase spaces (Figure 5) suggest that the control parameter N_Mot_/N_MT_ can to some extent compensate for and counteract the influence of the other control parameter v_g_/v_m_, also in agreement with our experimental observations (Figure 3G). Nematic networks can occur at low values of v_g_/v_m_, where polarity-sorting is favored, if N_Mot_/N_MT_ is small. This indicates that the absolute number of end-bound motors, and not only the relative amount, is a critical determinant of network fate.

In conclusion, we reduced the dimensionality of the organizational parameter space and identified two dimensionless control parameters. They characterize the collective forces produced by motor crosslinks that drives the formation of either nematic or polar networks of dynamic microtubules.

### HSET Organizes Microtubules with Natural Dynamics into Polar Networks

**Figure 6:**
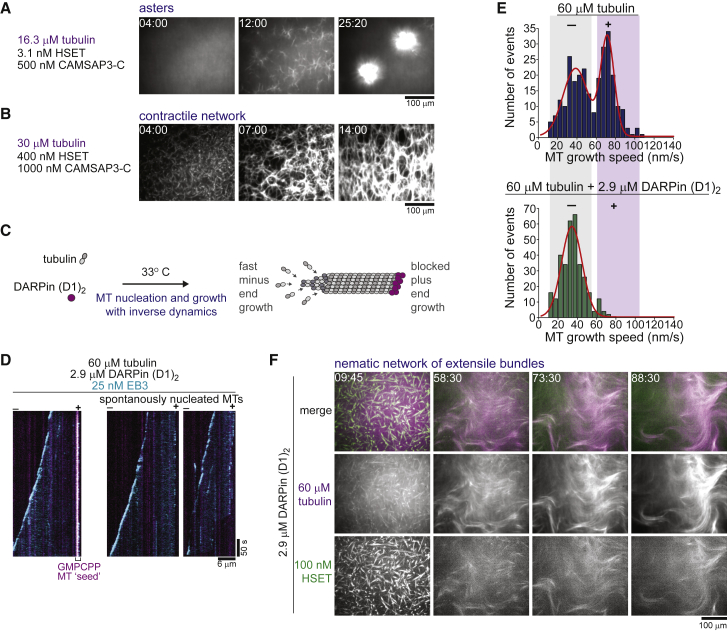
Microtubule Minus-End-Directed Motor HSET Organizes Asymmetrically Growing Microtubules into Asters and Nematic Networks of Extensile Bundles (A and B) Confocal fluorescence microscopy images showing time course of (A) HSET-mediated organization of microtubule asters and of (B) a globally contracting microtubule network of CAMSAP3-C-nucleated microtubules at the indicated protein concentrations. (C) Scheme showing inverted microtubule growth asymmetry in the presence of microtubule plus-end capper DARPin (D1)_2_. (D) Kymographs showing fast microtubule minus-end growth using Alexa546-EB3 to visualize microtubule ends growing at 60 μM tubulin from GMPCPP-stabilized microtubule “seeds” (left) or of spontaneously nucleated microtubules (right) in the presence of 2.9 μM DARPin (D1)_2_. (E) Microtubule growth speed distribution in the absence (top) and presence (bottom) of 2.9 μM DARPin (D1)_2_ at 60 μM tubulin. Number of microtubule growth episodes measured: without DARPin (D1)_2_, 271; with DARPin (D1)_2_, 328. (F) Confocal fluorescence microscopy images showing time course of mCherry-HSET-mediated organization of microtubules with inverted growth asymmetry into networks of extensile bundles in the presence of 2.9 μM DARPin (D1)_2_ at 60 μM tubulin. mCherry-HSET concentration was 100 nM. Temperature was 33°C. See also Figure S7 and Video S6.

Our understanding suggests that a minus-end-directed microtubule crosslinking motor such as kinesin-14 will have a strong tendency to form asters and accumulate at the centers of these asters when microtubule minus-ends are static, especially when the motor can become enriched between overlapping microtubules, as shown for kinesin-14 (Braun et al., 2017, Hentrich and Surrey, 2010). Self-organization experiments with purified human kinesin-14 HSET (Figure S1) and dynamic microtubules confirmed this expectation. They showed that over a range of tubulin (10–40 μM), CAMSAP3-C (250–1,000 nM), and HSET concentrations (3.1–400 nM), this motor formed either microtubule asters or contractile networks (Figures 6A and 6B). This has previously been observed for *X. laevis* kinesin-14 and clusters of *Drosophila* kinesin-14 with Taxol-nucleated microtubules, and for human HSET with microtubules growing from stabilized microtubule “seeds” (Hentrich and Surrey, 2010, Surrey et al., 2001).

### When Microtubule Dynamics Are Inverted HSET Forms a Nematic Network

**Figure S7:**
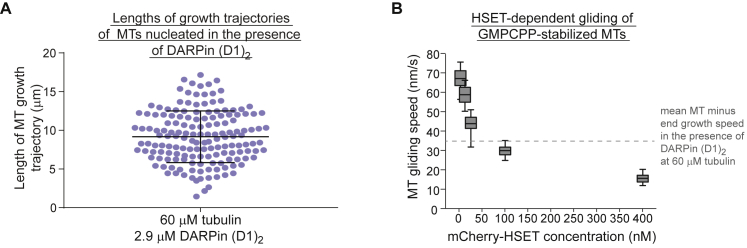
Microtubule Growth Episode Lengths in the Presence of DARPin and HSET Motor Speeds, Related to Figure 6 (A) Scatterplot depicting microtubule growth episode lengths at 60 μM tubulin in the presence of 2.9 μM DARPin (D1)_2_. Number of growth trajectories measured – 158. Horizontal lines indicate the mean and the standard deviation. (B) Box-and-whiskers plot depicting the dependence of HSET-driven microtubule gliding speeds on the mCherry-HSET concentration used to immobilise the motor on the glass surface for gliding assays with GMPCPP-stabilized microtubules. These speeds agree with previously measured vertebrate kinesin-14 gliding speeds (Braun et al., 2017, Hentrich and Surrey, 2010). The boxes extend from 25^th^ to 75^th^ percentiles, the whiskers extend from 5^th^ to 95^th^ percentiles, and the mean value is plotted as a line in the middle of the box. Number of gliding episodes measured at different mCherry-HSET concentrations: 3.1 nM – 160, 12.5 nM – 102, 25 nM – 129, 100 nM – 108, 400 nM – 92. All experiments were carried out in self-organization buffer at 33°C.

To test the generality of the rules of active network formation developed here, we asked whether HSET, a mitotic motor with a completely different domain structure and motile properties compared to kinesin-5, can also produce a nematic network instead of asters under the appropriate conditions. This should happen when the microtubule growth asymmetry is inverted so that HSET would have difficulty accumulating at the growing minus-ends. To engineer this condition, we replaced the minus-end stabilizer CAMSAP3-C by the designed ankyrin repeat protein (D1)_2_ (DARPin) that was previously shown to selectively inhibit microtubule plus-end growth (Figure 6C) (Pecqueur et al., 2012). We used a very high tubulin concentration to promote efficient spontaneous microtubule nucleation and to allow for fast minus-end growth. We verified that the DARPin selectively inhibited the growth of individual microtubule plus-ends under these conditions (Figures 6D and 6E), inverting the growth asymmetry. Fast minus-end growth reached the range of HSET-dependent gliding speeds (Figures 6E and S7).

Strikingly, under these conditions, HSET indeed produced nematic networks of extensile bundles with the motors now being evenly distributed within the dynamic network (Figure 6F; Video S6). This shows that, remarkably, both plus- and minus-end-directed motors can produce either locally contractile networks leading to asters or nematic networks of extensile bundles, depending on the experimental conditions. It validates our finding that motor directionality is not the sole determinant of its morphogenetic potential. Instead, relative motor speed compared to the growth speed of the microtubule end toward which it is directed, and the relative concentrations of motors and microtubules are the critical control parameters that determine the architecture of the forming filament network.

Video S6. Minus-end-Directed Kinesn-14 HSET (Green) Organizes Spontaneously Nucleated Dynamic Microtubules with Inverted Growth Asymmetry into Nematic Networks of Extensile Bundles, Related to Figure 6 Protein concentrations were: tubulin – 60 μM, DARPin (D1)_2_ – 2.9 μM, and mCherry-HSET – 100 nM. Time is in min:s. Imaging was carried out at 33°C.

## Discussion

We investigated the determinants of polar versus nematic cytoskeletal network organization using mitotic motor proteins and dynamic microtubules. We focused on these two prototypical active filament network states because of their importance for bipolar spindle organization required for chromosome segregation during cell division. Compared to previous self-organization assays (Hentrich and Surrey, 2010), several technical improvements (STAR Methods) allowed us to explore a considerably larger part of the phase space of network organizations than before, because a wider range of protein activities could be investigated. Using microtubules with tunable growth dynamics, we found that minus- and plus-end-directed microtubule crosslinking motors that in the cell typically contribute exclusively to either polar or nematic microtubule organizations (Gaetz and Kapoor, 2004, Heald et al., 1996, Mayer et al., 1999, Sawin et al., 1992), have nevertheless the general capacity to produce both types of networks. Our ability to reverse the natural preference of each motor for a particular organization by controlling the microtubule growth asymmetry demonstrates that the organizational capabilities of a motor can only be understood by also taking the dynamic properties of microtubules into account.

Nematic microtubule networks were previously observed only for rather artificial conditions compared to the situation in the central spindle (Henkin et al., 2014, Sanchez et al., 2012). Here, we found that nematic microtubule networks can also self-organize under closer-to-physiological conditions, reconstituting kinesin-5’s natural microtubule organizing function during spindle assembly. We showed that this state can be obtained with dynamic, and not only static microtubules as shown previously, and motor-dependent microtubule crosslinking at high microtubule densities is sufficient for generating this prototypical network state.

**Figure 7:**
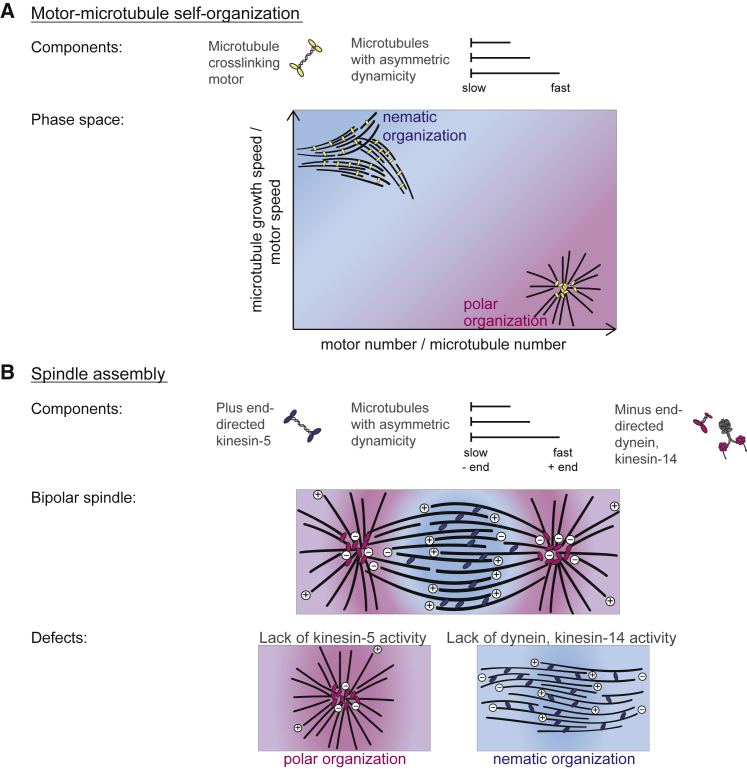
Summarizing Scheme of the Rules of Active Network Self-Organization (A) Key molecular components and the organizational phase space defined by the two identified control parameters that determine the outcome of microtubule/motor network self-organization. (B) Relevance of the control parameter-based rules for normal bipolar spindle assembly in cells and their consequences for the characteristic shapes of defective spindles after motor inactivation.

We identified two control parameters that determine microtubule/motor network organization and reflect underlying mechanistic driving forces; (1) the ratio of motor number per microtubule number that captures the organizational capacity of the system, and (2) the ratio of microtubule growth speed per motor speed that captures the competition between end-bound and side-bound motor crosslinks. Both control parameters combine a microtubule and a motor characteristic, emphasizing the importance of system-level properties for determining the outcome of active network self-organization. Together, these two control parameters define a phase space of reduced dimensionality that can be used to predict the organizational outcome of a microtubule/motor system (Figure 7A). Previous simulations of the special case of motor-mediated organization of static microtubules under a confining force produced a phase space that maps well onto our reduced dimensionality phase space, emphasizing the generality of its structure (Head et al., 2011).

The rules for microtubule/motor network organization derived here are independent of the directionality and detailed molecular domain structure of the motors, which differ between plus-directed kinesin-5 KIF11 and minus-directed kinesin-14 HSET (Fink et al., 2009, Kashina et al., 1996, Scholey et al., 2014). Hence, these rules are universally applicable for network organization by motors and dynamic microtubules. In the presence of crowding agents, fast microtubule growth speeds are not required for nematic organization (Henkin et al., 2014, Sanchez et al., 2012). This is likely the case because crowding-induced bundling strongly favors side-bound over end-bound motor crosslinks, even when microtubules are static.

Our work reveals that the asymmetry of microtubule growth properties is an important morphogenetic determinant in the spindle and responsible for motors of opposite directionality having different preferences for network organization. Fast and dynamically growing microtubule plus-ends and static minus-ends favor nematic versus polar network organization by plus- and minus-motors, respectively. This basic design principle puts a strong constraint on the structure of the bipolar spindle that can be conceptualized as a central nematic network coexisting stably with two polar networks (Figure 7B). Balanced motor activities, as well as localized microtubule nucleation around chromosomes, likely play an important role for mediating this coexistence (Brugués et al., 2012, Burbank et al., 2007).

We observed the gradual transition between nematic and polar organization in our experimental and simulated phase spaces. This may imply that, in the spindle, the control parameters can change gradually as the degree of microtubule polarity-sorting changes from spindle center to pole (Brugués et al., 2012). Our results show that 2- to 3-fold changes in protein concentrations can be sufficient to transition from nematic to polar organization, providing the cell with an opportunity to affect microtubule network architecture by spatially or temporally controlling protein activities.

Our rules for motor-mediated cytoskeletal network organization also provide explanations for several spindle phenotypes observed in mitotic cells or meiotic cell extract resulting from a variety of perturbations (Figure 7B). When plus-end directed motors like kinesin-5 dominate after dynein inhibition, a nematic network of aligned microtubules with unfocused poles forms (Gaetz and Kapoor, 2004, Heald et al., 1996). This is because microtubule plus-ends grow faster than the moderately fast kinesin-5, similar to the network formed by purified KIF11 and dynamic microtubules in our experiments at high tubulin concentrations. In contrast, when minus-directed motors like dynein dominate after kinesin-5 inhibition in mitotic/meiotic cytoplasm, monopolar spindles form in the absence of significant minus-end growth (Mayer et al., 1999, Sawin et al., 1992). This is similar to microtubule asters formed by purified minus-directed motors and microtubules with non-dynamic minus-ends.

The physiological importance of relative microtubule growth and motor speeds is also supported by the observation that an artificial kinesin-5 that is fast enough to accumulate at microtubule plus-ends has been shown to prevent normal spindle formation in meiotic cell extract by separating half-spindles, forming a central inverted pole instead of a nematic network (Cahu and Surrey, 2009).

Finally, the importance of the number of motors per microtubule for network organization may explain why a variety of perturbations that lead to a reduction of microtubule numbers (by either reducing microtubule nucleation efficiency or microtubule stability) without affecting motor abundance, disfavor the formation of the central nematic zone and hence induce monopolar (or multipolar) spindle phenotypes (Aguirre-Portolés et al., 2012, Cassimeris and Morabito, 2004, Hannak et al., 2002, Kline-Smith and Walczak, 2002, Petry et al., 2011).

Hence, the concepts developed here not only explain the contributions of asymmetrically growing microtubules and different motors for normal spindle shape, but also for commonly observed phenotypes when motor activities or the numbers of the major molecular constituents of the spindle network are unbalanced. The next challenge will be to reconstitute and model more complex active networks, extending the concepts developed here. A major aim will be to gain a quantitative understanding of the conditions allowing the unique coexistence of nematic and polar networks in multi-motor systems such as the bipolar spindle. Furthermore, it will be interesting to see to what extent these principles can also be extended to other cytoskeletal systems in cells such as dynamic actin networks (Goldstein et al., 2008, Palacios and St Johnston, 2002, Wollrab et al., 2018).

## STAR★Methods

### Key Resources Table

**Table:** 

REAGENT or RESOURCE	SOURCE	IDENTIFIER
**Bacterial and Virus Strains**		

Bacterial strain for molecular cloning: *Escherichia coli* DH5α	EMBL	Strain name: DH5α
Bacterial strain for generating bacmids: *Escherichia coli* DH10MultiBac	Gift from Imre Berger	Strain name: DH10MultiBac
Bacterial strain for recombinant protein expression: *Escherichia coli* BL21 pRil	EMBL	Strain name: BL21 pRil

**Chemicals, Peptides, and Recombinant Proteins**		

HSET	This study	Corresponding recombinant DNA: pCT012
mCherry-HSET	This study	Corresponding recombinant DNA: pJR291
KIF11-mGFP	This study	Corresponding recombinant DNA: pJR303
mGFP-CAMSAP3-C	This study	Corresponding recombinant DNA: pCT010
mCherry-CAMSAP3-C	This study	Corresponding recombinant DNA: pCT011
SNAP-EB3	Previously used by Jha et al. (2017)	N/A
DARPin (D1)_2_	Gift from Marcel Knossow and Andreas Plückthun; Pecqueur et al., 2012	N/A
Pig brain tubulin	Purified according to Castoldi and Popov (2003)	N/A
Catalase	Sigma-Aldrich	Cat#: C40
Glucose Oxidase	Serva	Cat#: 22778.01
Bovine Serum Albumin	Sigma-Aldrich	Cat#: 05470
Κ-casein	Sigma-Aldrich	Cat#: C0406
Β-casein	Sigma-Aldrich	Cat#: C6905
Neutravidin	LifeTechnologies	Cat#: A2666
(3-Glycidyloxypropyl)trimethoxy-silane	Sigma-Aldrich	Cat#: 440167
Biotin-CONH-PEG-NH_2_ (3000 Da)	Rapp Polymere Gmbh	Cat#: 133000-25-20
HO-PEG-NH_2_ (3000 Da)	Rapp Polymere Gmbh	Cat#: 103000-20

**Deposited Data**		

Source data for Figures 4, 5, S4, and S6.	This study	https://doi.org/10.17632/s8wz47nc9p.1

**Experimental Models: Cell Lines**		

Insect cells for recombinant protein expression: *Spodoptera frugiperda* 21 (Sf21)	EMBL	Cell line name: Sf21

**Recombinant DNA**		

pCT010 (pFastBacSTREP-mGFP-CAMSAP3-C)	This study	cDNA from Origene (NCBI Reference Sequence: NM_001080429.2)
pCT011 (pFastBacSTREP-mCherry-CAMSAP3-C)	This study	cDNA from Origene (NCBI Reference Sequence: NM_001080429.2)
pCT012 (pFastBacSTREP-HSET)	This study	cDNA from Origene (NCBI Reference Sequence: NM_002263.3)
pJR291 (pFastBacSTREP-mCherry-HSET)	This study	cDNA from Origene (NCBI Reference Sequence: NM_002263.3)
pJR303 (pFastBacSTREP-KIF11-mGFP)	This study	cDNA from ImaGene (GenBank ID: BC136474.1)
pETMZ-SNAP-EB3	First used by Jha et al. (2017)	Original cDNA gift from Michael Steinmetz; Montenegro Gouveia et al., 2010

**Software and Algorithms**		

FiJi for image analysis	NIH, USA	https://fiji.sc/
Python for data analysis	CWI, the Netherlands	https://www.python.org/
Wolfram Mathematica for data analysis	Wolfram Mathematica	https://www.wolfram.com/mathematica/
Cytosim	Nedelec and Foethke, 2007	https://github.com/nedelec/cytosim

**Other**		

StrepTrap HP column	GE Healthcare	Cat#: 28907547
HiPrep 26/10 Desalting column	GE Healthcare	Cat#: 17508701
Superose 6 Increase 10/300 GL column	GE Healthcare	Cat#: 29091596
Superose 6 XK 16/70 column	GE Healthcare	Cat#: 90100042

### Contact for Reagent and Resource Sharing

Further information and requests for resources and reagents should be directed to and will be fulfilled by the Lead Contact Thomas Surrey (thomas.surrey@crick.ac.uk).

### Experimental Model and Subject Details

*Escherichia coli* bacterial strains DH5α, BL21 pRil, and DH10MultiBac were grown in Luria Bertani (LB) medium in the presence of appropriate antibiotics.

For expression of recombinant proteins in insect cells we used *Spodoptera frugiperda* strain *Sf*21 grown in suspension at 27°C in Sf-900TM III SFM (1x) Serum Free Medium (GIBCO). Absence of mycoplasma contamination was verified regularly.

### Method Details

#### Molecular cloning

The full-length protein coding sequences of human kinesin-5 KIF11 (aa 1 – 1056) and human kinesin-14 HSET (aa 1 - 673), and the C-terminal fragment of human CAMSAP3 (CAMSAP3-C: aa 757 - 1276) were amplified by PCR using the respective cDNAs as templates (for KIF11 Genbank: BC136474.1 (ImaGene); for HSET NCBI reference Sequence: NM_002263.3 (Origene), for CAMSAP3-C NCBI Refernece Sequence: NM_001080429.2 (Origene)). The C-terminal fragment of CAMSAP3 was chosen based on its ability to preferentially bind to and stabilize the microtubule minus end and inhibit its growth (Hendershott and Vale, 2014, Jiang et al., 2014). The PCR-amplified coding sequences were then cloned into pFastBacSTREP-based baculovirus expression vectors (Roostalu et al., 2015) to generate the following expression constructs: StrepTagII-KIF11-A_3_G_5_-mGFP (pJR303) having KIF11 C-terminally fused to an alanine (A) – glycine (G) linker followed by monomeric GFP (green fluorescent protein (Snapp et al., 2003, Zacharias et al., 2002); StrepTagII-HSET containing HSET without a fluorescent tag (pCT012), StrepTagII-mCherry-G_5_A-HSET having HSET N-terminally fused to monomeric Cherry (Shaner et al., 2004) separated by a GA-linker (pJR291); StrepTagII-mCherry-G_5_A-CAMSAP3-C (pCT010) and StrepTagII-mGFP-G_5_A-CAMSAP3-C (pCT011) having the C-terminal fragment of CAMSAP3 N-terminally fused to either mCherry or mGFP separated by a GA-linker. The StrepTagII in these constructs could later be removed by Tobacco Etch Virus (TEV) protease cleavage. The final protein products are referred to as KIF11-mGFP, untagged HSET, mCherry-HSET, mCherry-CAMSAP3-C, and mGFP-CAMSAP3-C throughout the manuscript. All constructs were verified by sequencing. Baculovirus preparation and protein expression in *Sf*21 insect cells (*Spodoptera frugiperda*) were carried out according to manufacturer’s protocols (Bac-to-Bac system, Life Technologies).

The bacterial expression construct for producing a full-length human EB3 with an N-terminal hexa-histidine and SNAP-tag has been described elsewhere (Jha et al., 2017).

#### Protein purifications

*Sf*21 cells expressing recombinant mGFP-CAMSAP3-C or mCherry-CAMSAP3-C were resuspended in ice-cold CAMSAP3 lysis buffer (50 mM HEPES, 300 mM KCl, 5 mM MgCl_2_, 1 mM EDTA, 5 mM 2-mercaptoethanol (2-ME), pH 8.0) supplemented with protease inhibitors (Roche), DNase I (10 μg ml/ml, Sigma), and avidin (10 mg per liter of culture to capture biotin from insect cell media). Resuspended cells were lysed by douncing (40 strokes) and the lysate was clarified by ultracentrifugation (183,860 *g,* 45 min, 4°C). Clarified lysate was then passed through a StrepTrap HP column (GE Healthcare). The column was washed with CAMSAP3 lysis buffer containing 0.5 mM ATP and then with CAMSAP3 lysis buffer. The protein was eluted in CAMSAP3 elution buffer (50 mM HEPES, 300 mM KCl, 2 mM MgCl_2_, 1 mM EDTA, 50 mM arginine, 50 mM glutamate, 2.5 mM D-desthiobiotin, 5 mM 2-ME, pH 7.5) supplemented with protease inhibitors. The N-terminal StrepTagII was removed by overnight TEV protease cleavage on ice. The protein was then passed through HiPrep Desalting columns (GE Healthcare) to exchange the buffer to CAMSAP3 storage buffer (50 mM HEPES, 300 mM KCl, 2 mM MgCl_2_, 250 mM sucrose, 50 mM arginine, 50 mM glutamate, 5 mM 2-ME, pH 7.5). The protein was then run once more over a StrepTrapHP column to remove unspecifically binding contaminants. The flow-through was then subjected to size-exclusion chromatography using a Superose 6 Increase column (GE Healthcare) equilibrated in CAMSAP3 storage buffer. The mGFP-CAMSAP3_C or mCherry-CAMSAP3-C containing fractions were pooled, concentrated (Vivaspin 30,000 MWCO, Sartorius), ultracentrifuged (278,088 *g*, 10 min, 4°C), aliquoted, snap frozen, and stored in liquid nitrogen until use.

*Sf*21 cells expressing recombinant KIF11-mGFP were resuspended in ice-cold KIF11 lysis buffer (50 mM Na-phosphate, 300 mM KCl, 5 mM MgCl_2_, 10 mM 2-ME, 1 mM ATP, pH 7.5) supplemented with protease inhibitors, DNase I (10 μg ml/ml), and avidin (10 mg per liter of culture). Resuspended cells were lysed by douncing (40 strokes) and the lysate clarified by ultracentrifugation (183,860 *g,* 45 min, 4°C). Clarified lysate was then passed over a StrepTrap HP column. The column was washed with KIF11 lysis buffer and protein was eluted in KIF11 elution buffer (50 mM Na-phosphate, 300 mM KCl, 2 mM MgCl_2_, 2 mM D-desthiobiotin, 10 mM 2-ME, 0.1 mM ATP, pH 7.5) supplemented with protease inhibitors. The N-terminal StrepTagII was removed by overnight TEV protease cleavage on ice. The protein was then purified further by size-exclusion chromatography using a Superose 6 XK 16/70 column (GE Healthcare) equilibrated in KIF11 storage buffer (50 mM Na-phosphate, 300 mM KCl, 2 mM MgCl_2_, 10 mM 2-ME, 0.1 mM ATP, pH 7.5). The KIF11-mGFP containing fractions were pooled, concentrated (Vivaspin 30,000 MWCO), ultracentrifuged (278,088 *g*, 10 min, 4°C), aliquoted, snap frozen, and stored in liquid nitrogen until use.

HSET and mCherry-HSET were purified like CAMSAP3 proteins, however HSET lysis buffer (50 mM Na-phosphate, 300 mM KCl, 5 mM MgCl2, 1 mM EGTA, 5 mM 2-ME, 0.5 mM ATP, pH 7.5), HSET elution buffer (50 mM Na-phosphate, 300 mM KCl, 1 mM MgCl2, 1 mM EGTA, 2.5 mM D-desthiobiotin, 5 mM 2-ME, 0.1 mM ATP, pH 7.5) and HSET storage buffer (50 mM Na-phosphate, 300 mM KCl, 1 mM MgCl2, 1 mM EGTA, 5 mM 2-ME, 0.1 mM ATP, pH 7.5) were used instead of the corresponding CAMSAP3 buffers.

The SNAP-EB3 protein was expressed in *Escherichia coli* BL21 pRIL, purified, and labeled with SNAP-Surface AlexaFluor546 (NEB) (called Alexa546-EB3 from here on) and stored in EB3 storage buffer (50 mM Na-phosphate, 400 mM KCl, 5 mM MgCl_2_, 0.5 mM 2-ME, pH 7.2) as described recently (Jha et al., 2017).

Porcine brain tubulin was purified as described (Castoldi and Popov, 2003). Purified tubulin was recycled and labeled with Alexa647-*N*-hydroxysuccinimide ester (NHS; Sigma-Aldrich), CF640R-NHS (Sigma-Aldrich), or biotin-NHS (Thermo Scientific), as described previously (Hyman et al., 1991). Labeling ratios were kept relatively low (below 0.5) to preserve protein activities.

Purified recombinant DARPin (D1)_2_ was a kind gift from Marcel Knossow and Andreas Plückthun (Pecqueur et al., 2012).

Protein concentrations were determined by Bradford assay or by spectroscopy measurements (absorption at 280 nm) for tubulin. Concentrations refer to protein monomers (KIF11-mGFP, HSET and CAMSAP3-C constructs, Alexa546-EB3, DARPin (D1)_2_) or dimers (tubulin).

#### Microtubule self-organization assays with dynamic microtubules

Flow chambers were assembled from a glass slide and a cover glass separated by a double sticky tape. Unlike in previous self-organization studies with microtubules and motors, for improved surface passivation here both glasses were silanized and reacted with polyethylene glycol as described (Bieling et al., 2010) except that HO-PEG-NH_2_ (3000 Da) (Rapp Polymere) was used. This modification together with biochemical improvements allowed exploring a considerably wider range of protein activities than in previous self-organization experiments (Hentrich and Surrey, 2010). A flow chamber was washed with self-organization assay buffer (SAB: 20 mM PIPES, 1 mM EGTA, 2 mM MgCl_2_, 50 mM KCl, 1% glucose (w/vol), 1.5 mM ATP, 1 mM GTP, 5 mM 2-ME, pH 6.8) and warmed up to 33°C on a metal block. Meanwhile the final assay mix was prepared on ice and ultracentrifuged at 278,088 x *g* for 10 min at 4°C. The supernatant was transferred to a fresh Eppendorf tube, allowed to come to room temperature, and then flowed into the pre-warmed flow chamber on a 33°C metal block. The sample was then transferred to the spinning disc confocal microscope and imaging was started 2-4 min after flowing the sample into the warm chamber.

For KIF11-mediated microtubule organization in the presence of CAMSAP3-C, the final assay mix consisted of 64% SAB, 27.2% BRB80 containing oxygen scavengers, BSA and recycled and fluorescently labeled tubulin, 3.3% mCherry-CAMSAP3-C solution in CAMSAP3-C storage buffer and 5.5% KIF11-mGFP solution in KIF11-mGFP storage buffer. The final protein concentrations in the assay were 164 μg/ml catalase, 684 μg/ml glucose oxidase, 1 mg/ml BSA, 7.5 – 30 μM recycled and fluorescently labeled tubulin (containing 3.5 – 7% Atto647- or CF640R-labeled tubulin), 250 – 1000 nM mCherry-CAMSAP3-C, and 9 – 273 nM KIF11-mGFP, as indicated in the text.

For HSET-mediated microtubule organization in the presence of CAMSAP3-C, the final assay mix consisted of 63.5% SAB, 27.2% BRB80 containing oxygen scavengers, BSA and recycled and fluorescently labeled tubulin, 4.8% mGFP-CAMSAP3-C or mCherry-CAMSAP3-C solution in CAMSAP3-C storage buffer, and 4.5% mCherry-HSET solution in HSET storage buffer. The final protein concentrations in the assay were 164 μg/ml catalase, 684 μg/ml glucose oxidase, 1 mg/ml BSA, 10 – 30 μM recycled and fluorescently labeled tubulin (containing 3.5 – 7% Atto647- or CF640R-labeled tubulin), 250 – 1000 nM mGFP-CAMSAP3-C or mCherry-CAMSAP3-C, and 3.1 – 400 nM mCherry-HSET or untagged HSET.

For HSET-mediated microtubule organization in the presence of DARPin (D1)_2_ (leading to inverted microtubule dynamics), the final assay mix consisted of 50.5% SAB, 44% BRB80 containing oxygen scavengers, BSA and recycled and fluorescently labeled tubulin, 4.5% mCherry-HSET solution in HSET storage buffer, and 1% DARPin (D1)_2_ solution in DARPin (D1)_2_ storage buffer. The final protein concentrations in the assay were 164 μg/ml catalase, 684 μg/ml glucose oxidase, 1 mg/ml BSA, 60 μM recycled and fluorescently labeled tubulin (containing 3.5% CF640R-labeled tubulin), 2.9 μM DARPin (D1)_2_, and 100 nM mCherry-HSET.

#### Microtubule nucleation assays

The flow chamber assembly and all sample preparation steps were performed similarly to the self-organization assay except that imaging was performed now by total internal reflection (TIRF) microscopy and SAB contained 0.15% (w/vol) methylcellulose (SAB-MC, methylcellulose c_p_ 4,000, Sigma-Aldrich) to facilitate microtubule positioning near the coverslip for imaging.

To evaluate the effect of varying CAMSAP3-C concentrations on microtubule nucleation, we monitored the Alexa546-EB3 comets marking growing microtubule ends in a final assay mix consisting of 68% SAB-MC containing Alexa546-EB3, 27.2% BRB80 containing oxygen scavengers, BSA, recycled tubulin, and 4.8% mGFP-CAMSAP3-C solution in CAMSAP3-C storage buffer. The final protein concentrations in the assay were 164 μg/ml catalase, 684 μg/ml glucose oxidase, 1 mg/ml BSA, 30 μM recycled tubulin, 250 – 1000 nM mGFP-CAMSAP3-C, and 25 nM Alexa546-EB3.

To evaluate the effect of varying the tubulin concentration on microtubule nucleation we monitored fluorescently labeled microtubules in a final assay mix consisting of 68% SAB-MC, 27.2% BRB80 containing oxygen scavengers, BSA, recycled and fluorescently labeled tubulin, and 4.8% mGFP-CAMSAP3-C solution in CAMSAP3-C storage buffer. The final protein concentrations in the assay were 164 μg/ml catalase, 684 μg/ml glucose oxidase, 1 mg/ml BSA, 30 μM recycled and fluorescently labeled tubulin (containing 3.5% of CF640R-labeled tubulin), and 500 nM mGFP-CAMSAP3-C.

#### Microtubule dynamics assays

Flow chambers were assembled from a silanized and biotin-PEG functionalized cover glass (functionalized with a 9:1 mix of HO-PEG-NH_2_ (3000 Da) and biotin-CONH-PEG-NH_2_ (3000 Da), both Rapp Polymere) (Hentrich and Surrey, 2010) and a silanized and PEG-passivated (HO-PEG-NH_2_ (3000 Da)) counter glass prepared as described above for improved passivation, separated by double sticky tapes. The assay itself is a modification of the protocol developed earlier (Bieling et al., 2010). In short, the flow chamber was first washed on a metal block on ice with κ-casein buffer (SAB supplemented with 50 μg/ml κ-casein (Sigma-Aldrich)) and then incubated on a metal block on ice for 3 min in NeutrAvidin (LifeTechnologies) solution (50 μg/ml in κ-casein buffer). The chamber was subsequently washed with SAB and incubated for 3 min at room temperature with SAB containing an appropriate dilution of GMPCPP-stabilized biotinylated and fluorescently-labeled microtubule ‘seeds’ (prepared as described earlier) (Bieling et al., 2010). The chamber was then washed twice with SAB to remove the unbound ‘seeds’ followed by flowing in the final assay mix (see below) that had been brought to room temperature after first mixing it on ice, followed by ultracentrifugation at 278,088 x *g* for 10 min at 4°C. The flow chamber was then sealed with silicone grease. Imaging was started at 3 min and again at 60 min after placing the sample on the microscope stage.

For evaluating microtubule growth speeds in the presence of CAMSAP3-C, the final assay mix was composed of 68%SAB containing Alexa546-EB3, 27.2% BRB80 containing oxygen scavengers, BSA and recycled tubulin, and 4.8% mGFP-CAMSAP3-C solution in CAMSAP3-C storage buffer. The final protein concentrations in the assay were 164 μg/ml catalase, 684 μg/ml glucose oxidase, 1 mg/ml BSA, 25 nM Alexa546-EB3, 7.5 – 30 μM recycled tubulin, and 250 – 1000 nM mGFP-CAMSAP3-C, as indicated in the text.

For evaluating microtubule growth speeds in the presence of DARPin (D1)_2_, the final assay mix was composed of 50.5% SAB containing Alexa546-EB3, 44% BRB80 containing oxygen scavengers, BSA and recycled tubulin, 4.5% HSET storage buffer, and 1% DARPin (D1)_2_ solution in DARPin (D1)_2_ storage buffer. The final protein concentrations in the assay were 164 μg/ml catalase, 684 μg/ml glucose oxidase, 1 mg/ml BSA, 25 nM Alexa546-EB3, 60 μM recycled tubulin, and 2.9 μM DARPin (D1)_2_.

#### Microtubule gliding assays

Flow chambers were assembled from a poly-(L-lysine)-PEG (SuSoS)-passivated counter glass and an untreated cover glass separated by double sticky tape as described previously (Roostalu et al., 2011). The flow chamber was first washed twice with SAB, and then equilibrated for 2 min at room temperature in β-casein buffer (SAB containing 1 mg/ml β-casein (Sigma-Aldrich)), followed by incubation with either KIF11-mGFP (20.5 nM – 328 nM) or mCherry-HSET (3.125 nM – 400 nM) in β-casein buffer for 5 min on a metal block on ice to allow for unspecific immobilization of the motor to the untreated cover glass. The motors were pre-diluted in their own storage buffers (see above) to ensure identical incubation conditions at different motor concentrations. Unbound motor was subsequently removed by two washes with SAB at room temperature. Then the chamber was filled with the final assay mix composed of 72.8% SAB and 27.2% BRB80 (80 mM PIPES, 1 mM EGTA, 1 mM MgCl_2_, pH 6.8) containing oxygen scavengers, bovine serum albumin (BSA), and GMPCPP-stabilized fluorescently labeled microtubules (prepared as described previously (Roostalu et al., 2015), 3.5% CF640R tubulin). Final concentrations of accessory proteins in the assay mix were 164 μg/ml catalase (Sigma-Aldrich), 684 μg/ml glucose oxidase (Serva), and 1 mg/ml BSA (Sigma-Aldrich). The chamber was then sealed with silicone grease and transferred to the microscope. Imaging was started 2 minutes after placing the sample on a microscope stage.

#### Fluorescence microscopy

Microtubule self-organization assays were imaged either on a 3i Marianas spinning disc confocal fluorescence microscope described earlier (Baumann and Surrey, 2014), or on a Cairn spinning disk confocal system (Cairn Research, Faversham, UK) based on a Nikon Eclipse Ti frame equipped with an Andor Zyla sCMOS camera and X-light V2 spinning disk unit, always using a 20x objective. A 488 nm laser was used used to excite mGFP-, a 561 nm laser was used to excite mCherry- or Alexa546-, and a 638 nm laser was used to excite Alexa647-, or CF640R-labeled proteins. Multichannel time lapse imaging was performed by acquiring images at 20 - 45 s intervals as 4 - 5 z stacks spaced at 12.5 μm apart sequentially for each channel. Images were acquired at 5 s intervals for higher time resolution single channel time lapse experiments to visualize microtubule bundle extension or to follow bleach mark separation after bundle photobleaching by a high power laser pulse at 638 nm. Exposure times were between 200 – 300 ms for all laser lines. Individual z-planes are presented in the figures.

Microtubule gliding assays, microtubule dynamics assays, and microtubule nucleation assays were imaged by total internal reflection fluorescence (TIRF) microscopy either on an iMIC TIRF microsocope (FEI Munich) described elsewhere (Roostalu et al., 2015) or on a custom TIRF microscope (Cairn Research, Faversham, UK) based on a Nikon Ti-E frame described previously (Zhang et al., 2017) using a 100x objective. A 561 nm laser was used to excite mCherry- or Alexa546, and 638 nm or 640 nm lasers were used to excite Alexa647- and CF640R-labeled proteins. For multichannel time lapse imaging the images were acquired at 1-2 s intervals, imaging channels alternatively. Exposure times were between 100 – 200 ms for all channels.

Imaging conditions (laser power, acquisition frame rate and exposure time) were always kept constant within a set of experiments to allow for direct comparisons between samples. All imaging was performed in a heated chamber at 33°C ± 1°C.

### Quantification and Statistical Analysis

#### Microscopy image analysis

Fluorescence microscopy images were processed and analyzed in Fiji and in MATLAB. Raw TIRF microscopy images were aligned as described earlier (Maurer et al., 2014), and also corrected for microscope stage drift if necessary using the Image Stabilizer plugin for ImageJ (Kang Li, http://www.cs.cmu.edu/∼kangli/code/Image_Stabilizer.html). Microtubule growth speeds and microtubule gliding velocities were determined by kymograph analysis. A mean microtubule growth speed was the average of the speeds of individual growth episodes of Alexa546-EB3-marked microtubules (or mGFP-CAMSAP3-C marked microtubules at lower tubulin concentrations where high concentrations of mGFP-CAMSAP3-C decorated the microtubule lattice and prevented EB3 accumulation at the ends) observed in the evanescent field of the TIRF microscope (either from start of growth to catastrophe, and/or from the appearance of a EB3 comet in the evanescent field until its disappearance). This analysis provides only a rough estimate of microtubule growth speeds as microtubules frequently appear and then grow out of the evanescent field, and as opposed to surface-bound microtubules, can move around considerably even while growing in the evanescent field. The total length of microtubule growth trajectories for the nematic network regime was estimated from the same dataset by measuring the total EB3 comet displacement per microtubule by kymograph analysis (from the appearance of the EB3 comet in the evanescent field until its disappearance, or until the last frame of the kymograph). This analysis provides a lower limit of the actual microtubule lengths, because these measurements were conducted between 2-12 min after initiation of nucleation by temperature shift whereas the self-organization experiments were considerably longer in duration (up to 90 min) and likely contained longer microtubules. In addition, the EB3-marked growing microtubule ends frequently appear and then grow out of the evanescent field instead of staying near the glass surface throughout the whole microtubule lifetime. Mean microtubule gliding speeds were calculated as the average gliding speed of individual microtubules on the motor-coated glass surface. The total number of growth episodes, growth trajectories and microtubule gliding speeds measured for each condition are stated in the respective figure legends.

#### Simulation of active microtubule-motor networks in Cytosim

The model used is as previously described (Nedelec and Foethke, 2007). In brief, forces exerted on microtubules are due to motors connecting microtubules (crosslinks) and due to excluded volume interactions with other microtubules (Figure S3). Microtubule motion is determined by over-damped Langevin equations, describing Brownian dynamics in a viscous fluid. The simulation space is a thin cuboid with x, y and z-dimensions of size 40, 40 and 0.4 μm respectively. Periodic boundary conditions are enforced in the x and y-dimensions mimicking an unbounded space in the x-y plane. In the z-dimension microtubules are confined. Simulations were run for at least 6 times as long as the lifetime of the microtubule (50 mins), sufficient time for the topology of the simulated networks to stabilize (Figures 4A, 4B, and 4D, right).

Initially, a fixed number of randomly distributed microtubule ‘nucleators’ create microtubules at rate k_nuc_ with initial length L_0_. Dynamic instability of microtubule plus-ends is implemented with a two-state model without rescue defined by a constant catastrophe frequency k_cat_, a constant shrinkage speed v_s_ and a force-dependent growth speed v_g_ (Brun et al., 2009). The growth speed is reduced in the presence of an antagonistic force, f_a_ < 0, by an exponential factor, $e^{f_{a}/f_{g}}$, where f_g_ > 0 is a characteristic force. After a shrinking microtubule vanishes, its nucleator is free to nucleate again. Nucleation events and catastrophes are stochastic and generated as first-order random events with constant probability.

Excluded volume interactions between microtubules are implemented via a Hookean soft-core repulsive force such that they each had an effective volume of 0.02 μm^3^, described by an open-ended cylindrical shell with its long axis coincident with the microtubule and enclosed at either end by a hemisphere. The force between microtubules is $f_{s}=\kappa_{s}\left(d-d_{0}\right)$, for d < d_0_ and zero for d > d_0_, where d is the distance between microtubules, d_0_ is an equilibrium distance and $\kappa_{s}$ is a stiffness constant characterizing the strength of the steric force. The stiffness constant is large enough that excluded volume interactions dominate over thermal fluctuations but small enough that forces produced by crosslinking motors can cluster microtubule plus-ends. This choice was made to capture the effective behavior of short-range steric repulsion between microtubules. We omitted attractive interactions representing depletion forces used elsewhere (Letort et al., 2015), because they are not present in our experiments. Forces produced by steric interactions are always directed perpendicular to the microtubule axis, so as not to interfere with parallel sliding.

The motor KIF11 is modeled as a pair of motor domains connected by a Hookean spring-like link with resting length d_m_ and stiffness κ_m_. This link can rotate freely at both attachment points, such that the angle between two crosslinked microtubules is unconstrained. Diffusion of unbound motors is not modeled explicitly; it is assumed to be sufficiently fast that a uniform spatial distribution of unbound motors is maintained. If one motor of a pair is bound to a microtubule the other can bind to any microtubule within a range r_b_ at rate k_on_. Whereas motor binding and unbinding are stochastic events, bound motors move deterministically toward the plus-end of the microtubule at a speed which is linearly proportional to its load vector *f*_load_, given by $v=v_{m}\left(1+\underline{f_{\text{load}}}\cdot \underline{d}/f_{\text{stall}}\right)$, where *d* is a unit vector parallel to the microtubule, f_stall_ > 0 is a characteristic stall force and v_m_ > 0 is the unloaded speed of the motor. Motors detach from the microtubule side at a rate k_off_ and from the microtubule plus-end with a different rate k_end_, which are both modulated exponentially by the load on the motor and a characteristic unbinding force f_unbind_, according to Kramer’s law; $k=k_{\text{off}}\;exp\left(||\underline{f_{\text{load}}}||/f_{\text{unbind}}\right)$.

#### Quantification of extensile behavior in the nematic network state

To quantify the extensile behavior of the nematic state the parameter $<\hat{v}\cdot \hat{p}>$ was used (Figures S4C and S4D). The unit vector of the microtubule velocity, $\hat{v}$, was calculated as $\hat{v}=v/\left|v\right|$, where $v=\left(x_{-}\left(t+\text{Δ}t\right)-x_{-}\left(t\right)\right)/\text{Δ}t$ and $x_{-}\left(t\right)$ is the position of the microtubule minus-end at time *t*. The microtubule’s unit direction vector $\hat{p}$ points along the microtubule’s axis toward the plus-end at time *t+Δt*. All microtubules present in frames *t* and *t+Δt* were used in the average $<\hat{v}\cdot \hat{p}>$. For the data shown in Figure S4D, *Δt* = 100 s. Time-points after 25 min were analyzed by which time the microtubule population had reached its steady-state average length. The velocity was measured from the microtubules’ minus ends, in order to avoid a trivial contribution from the microtubules’ plus-end growth. The major contribution to $<\hat{v}\cdot \hat{p}>$ comes from anti-parallel sliding which moves microtubules backward as motors move toward their plus-ends (Figures S4B and S4C) and results in a negative value of $<\hat{v}\cdot \hat{p}>$ (Figure S4D). As the maximum motor speed is decreased from 30 nm/s (corresponding to the nematic network) to zero, the magnitude of $<\hat{v}\cdot \hat{p}>$ decreases (Figure S4D), but it does not vanish entirely at zero motor speed. This is because growing microtubule plus-ends push on the surrounding network, which can drive the minus ends backward. However, the resulting (negative) contribution to $<\hat{v}\cdot \hat{p}>$ remains small compared to the contribution from motor-induced sliding.

#### Parameter selection and phase space collapse

We adopted values measured from our experiments for critical parameters of our model (microtubule growth speed and motor speed) and otherwise used measured values from literature where possible (see Table S1). The dimensions of the x-y plane (40 × 40 μm) of the three-dimensional simulation space were chosen to be large compared to the average simulated microtubule length (2.5 μm).

In scanning the parameter space to identify control parameters we varied key parameters over reasonable ranges. The number of motors was explored up to a maximum of 16 motors per microtubule. The microtubules in the three phase spaces in Figure 5A cover a volume fraction of the simulation space ranging from 10 to 30%. Higher densities were explored (Figures 4A and S4A, top) up to a volume fraction of 70%.

#### Classification of active microtubule-motor networks

Two parameters were defined to classify the simulation outcomes and capture the gradual transition between the two distinct network types; the nematic network and the asters. Both parameters are derived from the motor crosslinks made between microtubules; V links connecting two microtubule plus-ends, T links connecting a microtubule plus-end to the side of another microtubule, H_p_ links connecting parallel microtubules sides at an internal angle of 0 ≤ θ < π/3, H_ap_ links connecting anti-parallel microtubules sides at an internal angle 2π/3 < θ ≤ π and X links connecting microtubule sides at an internal angle of π/3 ≤ θ ≤ 2π/3 (Figure 4C, left).

The first parameter, c_max_, is used to determine whether an aster is present in the network; it is the size of the largest cluster of microtubules connected via V-links, C_max_, as a proportion of the total number of microtubules N_MT_, i.e., c_max_ = C_max_/N_MT_. Simulated networks with c_max_ ≥ 0.01 were classified as asters and colored dark red in the simulated phase spaces (Figures 5, S5, and S6A). This threshold was chosen so that asters were dense enough to have a radially isotropic distribution of microtubules, visually similar to the experimental asters.

If the simulated network is not classified as an aster a second measure is used to identify the nematic network and characterize the transition in the phase space from the nematic network to the polar aster state. We defined the measure, P, quantifying the degree of polarity-sorting as; $P={\bar{\text{H}}}_{\text{p}}/\left({\bar{\text{H}}}_{\text{p}}+{\bar{\text{H}}}_{\text{ap}}\right)$ where ${\bar{\text{H}}}_{\text{p}}$and ${\bar{\text{H}}}_{\text{ap}}$ denote respectively the numbers of parallel and anti-parallel crosslinks, averaged over the final 4% of the simulated time. With this measure, a nematic network of crosslinked microtubules with totally mixed polarity would have P = 0.5 and a totally polarity-sorted network would have P = 1. In the simulated phase spaces (Figures 5, S5, and S6A) the parameter P was visualized as a color gradient from blue (P = 0.5) to red (P = 1).

To test the reproducibility of the simulations and our classification procedure we repeated simulations for entire phase spaces and observed that the classifications did not change significantly (data not shown).

#### Theoretical motor distribution profile along a growing microtubule

The mechanistic principle underpinning the critical model parameter v_g_/v_m_ can be explained through mathematical analysis of the motor distribution profile along a single growing microtubule (Figure S6B), assuming: (1) A microtubule of finite length growing with velocity v_g_. (2) Motor movement along MTs with a constant velocity v_m_. (3) Binding and unbinding of motors to the side of the microtubule with rate ${\text{k}}_{\text{on}}^{\text{'}}$ (molecules per unit length per time) and k_off_ respectively. (4) Motors that reach the plus-end of the microtubule do not detach immediately but remain bound and unbind at a rate k_end_. Analytical results for a more general version of this model based on a continuous approximation of the classic TASEP model of a driven lattice gas (Kruse and Sekimoto, 2002, Parmeggiani et al., 2004) have been reported previously (Tischer et al., 2010). In our model, the density of motors on the microtubule side, ρ(x,t), defined for 0<x, follows the equation,

(S1) $$\partial_{t}\rho =k_{on}^{'}\text{Θ}\left(v_{g}t-x\right)-k_{\text{off}}\rho -v_{m}\partial_{x}\rho$$

where the step function Θ(…) allows motors to bind only to existing positions on the microtubule side, since $\text{Θ}\left(v_{g}t-x\right)\;=1$ for $x<v_{g}t$ and is zero otherwise. The total number of side-bound motors, $n_{s}\left(t\right)$, is then given by

(S2) $$n_{s}\left(t\right)=\rho_{0}\left(L\left(t\right)-\lambda_{m}\left(1-e^{-\frac{L\left(t\right)}{\lambda_{m}}}\right)\right),$$

where $\rho_{0}=k_{on}^{'}/k_{\text{off}}$ and $\lambda_{m}=v_{g}/k_{\text{off}}.$ The total number of end-bound motors, $n_{e}\left(t\right)$, can be found by balancing fluxes at the microtubule’s plus-end,

(S3) $$\partial_{t}n_{e}\left(t\right)=-k_{\text{end}}n_{e}\left(t\right)+\left(v_{m}-v_{g}\right)\rho \left(v_{g}t,t\right).$$

The first term on the RHS accounts for unbinding of motors from the tip of microtubules and the second term accounts for the incoming flux from the microtubule side. With the initial condition $n_{e}\left(t\right)=0$ Equation S3 can be solved, see Tischer et al. (2010), to give,

(S4) $$n_{e}\left(t\right)=n_{\infty }\left(1-\psi e^{-\frac{L\left(t\right)}{\lambda_{m}}}-\left(1-\psi \right)e^{-\frac{L\left(t\right)}{\lambda_{e}}}\right),$$

where $n_{\infty }=\rho_{0}/k_{\text{end}}\left(v_{m}-v_{g}\right)$ is the number of motors at the end of an infinitely long microtubule, $\lambda_{e}=v_{g}/k_{\text{end}}$ and $\psi ={\left(1-\lambda_{e}/\lambda_{m}\right)}^{-1}$ is a factor controlling which of the transient terms in Equation S4 dominate the evolution of $n_{e}\left(t\right)$.

Dividing Equation S4 by Equation S2 one can write,

(S5) $$\frac{n_{e}\left(t\right)}{n_{s}\left(t\right)}=\frac{\left(v^{'}-1\right)\left(1-\psi e^{-\frac{k_{\text{off}}t}{v^{'}}}-\left(1-\psi \right)e^{-k_{\text{end}}t}\right)}{k_{\text{end}}\left(t-\frac{v^{'}}{k_{\text{off}}}\left(1-e^{-\frac{k_{\text{off}}t}{v^{'}}}\right)\right)}\text{for}\;\text{t}>0,$$

where $v^{'}=v_{m}/v_{g}$ and $\psi ={\left(1-v^{'}\left(k_{\text{off}}/k_{\text{end}}\right)\right)}^{-1}$. We see that the critical parameter $v_{m}/v_{g}$, which was found to be a control parameter in our model system (Figure 5D), sets the ratio of end-bound to side-bound motors on a single filament at time t, if the unbinding rates k_off_ and k_end_ are fixed. Competition between these two populations of motors is therefore a key mechanism driving motor and microtubule organization.

### Data and Software Availability

The computational model was implemented in Cytosim, publicly available at https://github.com/nedelec/cytosim. The Cytosim version specific to this study with additional code is available at https://github.com/nedelec/cytosim/commit/1e9b8be78dc8aacf3aea905f46f9bb4751665592. Source data (simulation configuration files and documentation) relating to Figures 4, 5, S4, and S6 can be found online at https://doi.org/10.17632/s8wz47nc9p.1.

## Supporting Citations

The following references appear in the Supplemental Information: Dogterom and Yurke, 1997, Leduc et al., 2004, Loughlin et al., 2010, Nogales et al., 1999, Schnitzer et al., 2000, Valentine et al., 2006, Walker et al., 1988.

## Supplemental Information

Table S1. Simulation Parameters, Related to STAR Methods
