# Supplementary material for: Determinants of Polar versus Nematic Organization in Networks of Dynamic Microtubules and Mitotic Motors
Source: Cell. 2018 Oct 18;175(3):796–808.e14. doi: 10.1016/j.cell.2018.09.029 (PMC6198040; doi:10.1016/j.cell.2018.09.029)
Supplement: Table S1. Simulation Parameters, Related to STAR Methods [file mmc1.pdf]

Cell, Volume *175*

Supplemental Information

Determinants of Polar versus Nematic Organization in Networks of Dynamic Microtubules and Mitotic Motors

Johanna Roostalu, Jamie Rickman, Claire Thomas, François Nédélec, and Thomas Surrey

TABLE S1. Related to STAR Methods.

**Parameter**	**Value**	**Note**
Simulation		
Time step	0.01 s	Computational parameter. Smaller time step gave the same result.
Total time simulated	50 mins	Similar to experiments
Geometry	40 x 40 x 0.4 µm	Periodic boundary conditions in x and y-dimension. Confinement in z-dimension.
Experimental chamber		
Viscosity	0.2 pN s/µm^2	ref (Letort et al., 2015)
Microtubule		
Rigidity	30 pN µm^2	ref (Dogterom and Yurke, 1997)
Steric radius	0.05 µm	ref (Nogales et al., 1999)
Steric force constant (κ_s)	50 pN/µm	Constrained by strength of thermal fluctuations and force of motors.
Average length (L)	2.5 µm	
Growth rate (v_g)	0.005- 0.03 µm/s	Measured in this study.
Shrinkage rate (v_s)	0.5 µm/s	ref (Walker et al., 1988)
Catastrophe rate (k_{cat})	0.00067 - 0.012 /s	Calculated as v_g /L.
Sensitivity of growth to force (f_g)	1.67 pN	ref (Dogterom and Yurke, 1997)
Nucleation rate (k_{nuc})	0.1 /s	Time for all microtubules to nucleate is short relative to total simulation time.
Motor		
**Motor domain**		
Stall force (f_{stall})	5 pN	ref (Valentine et al., 2006)
Unbinding force (f_{unbind})	5 pN	ref (Schnitzer et al., 2000)
Maximum motor speed (v_m)	0.03 µm/s	Measured in this study.
Binding radius (r_b)	0.1 µm	Twice the steric radius so motors can bind neighbouring microtubules.
Binding rate (k_{on})	5 /s	ref (Leduc et al., 2004)
Unbinding rate from side (k_{off})	0.1 /s	From run length = 0.3 µm, (Kapitein et al., 2005)
Unbinding rate from end (k_{end})	0.1 /s	ref (Surrey et al., 2001)
**Dual motor complex**		
Resting length (d_m)	0.08 µm	ref (Kashina et al., 1996)
Link stiffness (κ_m)	100 pN/µm	ref (Loughlin et al., 2010)
**Discretization parameters**		
MT section length	1 µm	For bending elasticity.
MT initial length (L_0)	0.025 µm	Length of MTs when nucleated.
MT minimum length	0.02 µm	Shorter MTs are deleted.
